# Identification of a Type IV-A CRISPR-Cas System Located Exclusively on *IncHI1B/IncFIB* Plasmids in *Enterobacteriaceae*

**DOI:** 10.3389/fmicb.2020.01937

**Published:** 2020-08-12

**Authors:** Enas Newire, Alp Aydin, Samina Juma, Virve I. Enne, Adam P. Roberts

**Affiliations:** ^1^UCL Eastman Dental Institute, University College London, London, United Kingdom; ^2^Centre for Clinical Microbiology, Royal Free Hospital, University College London, London, United Kingdom; ^3^Department of Tropical Disease Biology, Liverpool School of Tropical Medicine, Liverpool, United Kingdom; ^4^Centre for Drugs and Diagnostics, Liverpool School of Tropical Medicine, Liverpool, United Kingdom

**Keywords:** Type IV, *IncFIIK*, *IncFIB(K)*, inter-plasmid competition, mobile genetic element

## Abstract

Clustered Regularly Interspaced Short Palindromic Repeats (CRISPR) are diverse immune systems found in many prokaryotic genomes that target invading foreign DNA such as bacteriophages and plasmids. There are multiple types of CRISPR with arguably the most enigmatic being Type IV. During an investigation of CRISPR carriage in clinical, multi-drug resistant, *Klebsiella pneumoniae*, a Type IV-A3 CRISPR-Cas system was detected on plasmids from two *K. pneumoniae* isolates from Egypt (isolated in 2002–2003) and a single *K. pneumoniae* isolate from the United Kingdom (isolated in 2017). Sequence analysis of all other genomes available in GenBank revealed that this CRISPR-Cas system was present on 28 other plasmids from various *Enterobacteriaceae* hosts and was never found on a bacterial chromosome. This system is exclusively located on *IncHI1B/IncFIB* plasmids and is associated with multiple putative transposable elements. Expression of the *cas* loci was confirmed in the available clinical isolates by RT-PCR. In all cases, the CRISPR-Cas system has a single CRISPR array (CRISPR1) upstream of the *cas* loci which has several, conserved, spacers which, amongst things, match regions within conjugal transfer genes of *IncFIIK/IncFIB(K)* plasmids. Our results reveal a Type IV-A3 CRISPR-Cas system exclusively located on *IncHI1B/IncFIB* plasmids in *Enterobacteriaceae* that is likely to be able to target *IncFIIK/IncFIB(K)* plasmids presumably facilitating intracellular, inter-plasmid competition.

## Introduction

Clustered Regularly Interspaced Short Palindromic Repeats (CRISPR-Cas) are widespread, adaptive, RNA-mediated, immune systems found in the genomes of prokaryotic organisms (bacteria and archaea) that target invading foreign DNA such as bacteriophages and conjugative plasmids (Barrangou et al., 2007; Marraffini and Sontheimer, 2010a). CRISPR functions through a three-stage process: adaptation involving the acquisition of foreign DNA molecules as spacers, expression and maturation of the short CRISPR RNAs (crRNAs), and the interference with a cognate invading foreign DNA molecule (Rath et al., 2015). The classification of CRISPR-Cas systems is continuously updated to include newly identified subtypes. To date, CRISPR-Cas systems are classified into two classes, six Types (I–VI), and ∼ 33 subtypes (Koonin et al., 2017; Makarova et al., 2018, 2020). There is ongoing discovery of multiple, novel class 2 CRISPR-Cas systems (Makarova et al., 2020). The two classes differ according to the effector module; class 1 utilizes multi-protein effector Cas complexes, while class 2 utilizes a single-protein effector [Type II contains Cas9; Type V contains Cas 12a (previously known as Cpf1), Cas12b (previously known as C2c1), Cas12c (previously known as C2c3), Cas12d (previously known as CasY), and Cas12e (previously known as CasX); and Type VI contains Cas13a (previously known as C2c2), Cas13b, and Cas13c] (Makarova and Koonin, 2015; Makarova et al., 2015, 2017; Pyzocha and Chen, 2018). CRISPR-Cas systems are confirmed, or expected, to provide immunity against viruses and other mobile genetic elements (MGEs), except for transposon-encoded CRISPR-Cas systems that lack the interference module and therefore are predicted to perform functions distinct from adaptive immunity (Makarova et al., 2020). Most of the CRISPR types target DNA, some types specifically target RNA such as Type VI, while Type III CRISPR systems are unique because they exhibit both RNA interference and DNA interference *in vivo* to protect their microbial hosts (Marraffini and Sontheimer, 2008, 2010b; Hale et al., 2009; Deng et al., 2013; Manica et al., 2013; Goldberg et al., 2014; Tamulaitis et al., 2014; Zebec et al., 2014; Peng et al., 2015; Samai et al., 2015; Elmore et al., 2016; Estrella et al., 2016; Kazlauskiene et al., 2016; Zhang and Ye, 2017; Ozcan et al., 2019; Lin et al., 2020).

**FIGURE 1:**
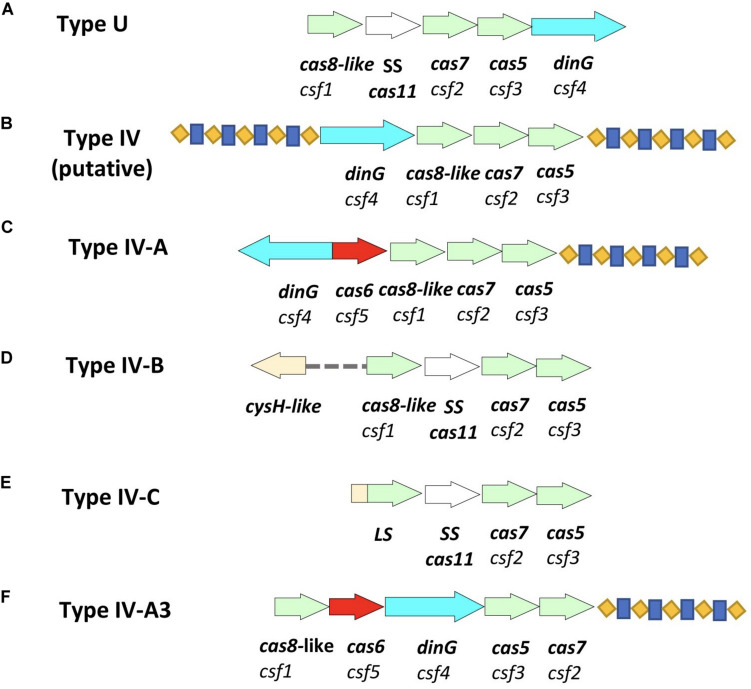
Schematic representation of Type IV CRISPR-Cas systems and the newly identified variant of Type IV-A. **(A)** Type U (unknown) as identified in 2013 (Makarova et al., 2013). **(B)** Type IV (putative) as identified in *Acidithiobacillus ferrooxidans* in 2015 (Makarova et al., 2015), however, the two associated repeat-spacer arrays were identified in this study. **(C)** Type IV-A identified in *Thioalkalivibrio sp.* K90mix (TK90_2699-TK90_2703) in 2017 (Makarova et al., 2018). **(D)** Type IV-B identified in *Rhodococcus jostii* RHA1 (RHA1_ro10069-RHA1_ro10072) in 2017 (Makarova et al., 2018). **(E)** Type IV-C identified in *Thermoflexia bacterium* D6793_05715-D6793_05700 (Makarova et al., 2020). **(F)** Type IV-A3 as detected in *Enterobacteriaceae* isolates and genomes in this study. Arrows in different colors represent genes; red represents *cas6*; bright blue represents *dinG*; light green represents other essential genes of the system; *cas8*-like/LS, *cas7* and *cas5*; white represents *cas11;* blue-yellow pattern represents the direct repeat-spacer loci.

Type IV was previously called the Unknown Type (Type U), due to its rare occurrence and lack of the adaptation module, until an updated classification in 2015 (Makarova et al., 2013; Zhang and Ye, 2017). It was then named Type IV (putative) after its identification in *Acidithiobacillus ferrooxidans* presenting a different genetic arrangement of Type U *cas* genes (Makarova et al., 2015). In 2017, Type IV classification was updated, after its identification in *Thioalkalivibrio sp.* K90mix (TK90_2699-TK90_2703), to show an associated repeat-spacer array for a *cas* loci that have *csf4* (*dinG*), *csf5* (*cas6-Like*), *csf1* (*cas8-Like*), *csf2* (*cas7*), and *csf3* (*cas5*) genetic arrangement, respectively, and was then assigned as Type IV-A (Koonin et al., 2017). In 2018, a variant of Type IV that lacks a repeat-spacer array from *Rhodococcus jostii* RHA1 (RHA1_ro10069-RHA1_ro10072), was assigned as Type IV-B (Figure 1; Makarova et al., 2018). In 2019, the Type IV-C CRISPR-Cas system was formally classified as a distinct subtype after its identification in nine contigs; mostly from thermophilic microorganisms (Makarova et al., 2020). Other papers have also proposed the classification of Type IV-D, Type IV-E, and subgroups of Type IV-A(1-4) (Crowley et al., 2019; Pinilla-Redondo et al., 2019), however, the suggested subgroups did not have a unified genetic arrangement corresponding for each of the named Type IV-A(1-4) variants. Type IV CRISPR-Cas systems were shown to employ crRNA-guided effector complexes (Ozcan et al., 2019). Type IV is the only type to possesses *csf4* (*dinG*) in its CRISPR-Cas loci (Dwarakanath et al., 2015; Koonin et al., 2017), and it was recognized initially as the signature proteins for Type IV systems, (Makarova and Koonin, 2015; Crawley et al., 2018) although recently subtype IV-D has been shown to carry a helicase of the RecD family in place of the archetypal DinG (Pinilla-Redondo et al., 2019). To date, Type IV variants (IV-A, IV-B, and IV-C) described above show different genetic arrangements and orientation of *cas* loci, and they all lack the adaptation module. Also, all Type IV CRISPR-Cas systems are encoded by bacterial plasmids, prophages or other, uncharacterized integrated elements (Faure et al., 2019b). Thus, it has been hypothesized that Type IV is similar to an ancestral innate immune system that gained adaptive ability by associating with a transposon-like element containing *cas1* and *cas2* (Rath et al., 2015).

## Materials and Methods

### Clinical Isolates Sequencing

Three clinical isolates were investigated; *Klebsiella pneumoniae-53* and *K. pneumoniae-65* were isolated from Egyptian university teaching hospitals (2002–2003), and *K. pneumoniae-CR5* from University College London Hospital in the United Kingdom (2017). The bacterial genomic DNA sequencing was conducted at MicrobesNG (Birmingham, United Kingdom). Isolates were sequenced using an Illumina HiSeq 2500 and an Illumina MiSeq instruments, to boost coverage, with a 2 × 250 bp paired end sequencing using Nextera XT library prep.

### CRISPR-Cas System Identification and Characterization

DNA sequences were analyzed using CRISPRFinder, CRISPRCasFinder, CRISPRTarget, and Snapgene (GSL Biotech) (Grissa et al., 2007; Lundgren et al., 2015; Couvin et al., 2018; GSL, 2020). The Cas domain analyses were performed by HHpred (Sensitive protein homology detection, function, and structure prediction based on HMM–HMM comparison) at MPI bioinformatics Toolkit^1^ (Zimmermann et al., 2018). HHpred was performed using NCBI_Conserved_Domains(CD)_v3.16 and TIGRFAMs_v15.1 databases and Bac_Escherichia_coli_K12_07_Mar_2017 proteome settings. Multi-Locus Sequence Typing, resistance genes and plasmids were identified using MLST, ResFinder, and PlasmidFinder, respectively (Carattoli et al., 2014). Spacer analysis was performed by BLAST and Geneious (Kearse et al., 2012). A phylogenetic UPGMA-based tree was constructed for CRISPR arrays and Cas proteins using MEGA X 10.1 (Kumar et al., 2016; Shen et al., 2017). The alignment of the regions containing protospacers (including 10 bp flanking the protospacer) associated with CRISPR1 repeats were investigated to identify putative protospacer adjacent motif (PAM) signature, as described in Pinilla-Redondo et al. (2019). PAMs were identified based on compared alignments of nucleotides immediately preceding each detected protospacer in all or up to ten unique protospacers of all the investigated sequences. The leader sequence was identified by sequence alignment. Direct repeats and PAM conservation were assessed using WebLogo, RNA secondary structure was predicted using RNAfold (Hofacker, 2003; Crooks et al., 2004; Mathews, 2005; Mojica et al., 2009). The GC skew plots were generated using the GenSkew online analysis tool^2^. Presence in other GenBank sequences was investigated by BLASTn^3^.

### CRISPR-Cas System Expression

The CRISPR-Cas loci expression was tested. RT-PCR was performed using LightCycler^®^ RNA Amplification Kit SYBR Green (Roche Diagnostics Ltd., United Kingdom). The primers were designed for fully characterized genes (*csf2*-fw:AAAATGCGGTCTCAACTTCCG; *csf2*-rev:TGACGAAGAG TTCCCCGAATG), (*dinG*-fw:GAGTCTGCCGGATTGTCGTTA; *dinG*-rev:GTACCAGATAGCCCAGCGTTT), and (*cas6*-fw:AAT GCGTTTCGGTTGCGTATC; *cas6*-rev:GAGTACGGCAGCTT CTCTCC).

## Results and Discussion

### Identification of Type IV-A3 CRISPR-Cas in Clinical and GenBank Isolate Sequences

Type IV-A-3 CRISPR-Cas, based on the gene composition and genetic architecture of the IV-A variants detected in *K. pneumoniae* as described in Pinilla-Redondo et al. (2019), was detected on a total of thirty-one (three clinical isolates and twenty eight sequences from GenBank) *IncHI1B/IncFIB(Mar)* plasmid sequences within *Enterobacteriaceae* (Figure 1 and Supplementary Table S1). The *IncH1B*/*IncFIB* plasmids are large, low copy number, conjugative plasmids with narrow-host-ranges, which are found in multiple genera of *Enterobacteriaceae* (Zhong et al., 2005; Suzuki et al., 2010; Faure et al., 2019a). An important feature of *IncH1B*/*IncFIB* plasmid biology is the entry exclusion by which the cells that contain an *IncF*/*IncH* plasmid become poor recipients in additional conjugation rounds (Garcillan-Barcia and de la Cruz, 2008; Ravenhall et al., 2015); which frees a resident plasmid from competition with related plasmids at segregation during bacterial division but may contribute to limiting plasmid dissemination among potential hosts.

This Type IV-A3 CRISPR-Cas is characterized by the presence of a *cas* loci containing *dinG*, which is a distinct feature of Type IV-A CRISPR-Cas system that was shown to be a requirement for the system functional activity in *Pseudomonas aeruginosa* (Crowley et al., 2019; Pinilla-Redondo et al., 2019; Makarova et al., 2020), a conserved leader sequence and a CRISPR array in all the detected sequences and they all show homology to each other. These Type IV-A3 CRISPR-Cas systems were initially detected by BLAST that confirmed the presence of three genes; *cas7*, *dinG*, and *cas6*, and further HHpred analysis of other associated ORFs revealed the presence of two more genes; *cas5* and *cas8-like*.

### Association Between Type IV-A3 CRISPR-Cas Sequences and *IncHI1B/IncFIB(Mar)* Plasmids

We also identified partial related Type IV-A3 systems either (*cas8*-like, *cas6*, and *dinG*) or (*cas7* and a CRISPR array) occurring on other *IncHI1B/IncFIB(Mar)* plasmids (Supplementary Table S1). Partial and complete Type IV-A3 system characterization showed occurrence of a range of different IS elements and retrotransposons (group II introns) (Supplementary Table S1). The average GC content of this Type IV-A3 CRISPR-Cas loci (47.7 ± 0.01%) was found to be closer to that of the *IncHI1B/IncFIB(Mar)* plasmids on which they reside (46.2 ± 0.01%), compared to the chromosomal sequences of the bacterial host (57 ± 0.02%), (Table 1).

**TABLE 1:** Comparison of the GC content of the CRISPR, host plasmid and host strain chromosome.

	Sequence (strain name, plasmid name, Accession number)	Chromosomal Size (bp)	Chromosomal GC Content (%)	Plasmid Size (bp)	Plasmid GC Content (%)	New Type IV-A Size (bp)	New Type IV-A GC Content (%)
1	*K. pneumoniae* 234-12, pKpn23412-362, CP011314.1	5,278,254	57	361,964	48	5,671	47
2	*K. pneumoniae* Kp15, pENVA, HG918041.1	*	*	253,984	47	6,093	47
3	*E. coli* strain Ecol_422, pEC422_1, CP018961.1	4,747,607	51	289,903	46	5,864	48
4	*K. pneumoniae* 825795-1, unnamed1, CP017986.1	5,373,055	51	244,706	45	5,979	48
5	*K. pneumoniae* KP_Goe_828304, pKp_Goe_304-1, CP018720.1	5,373,056	58	246,757	45	6,034	48
6	*K. pneumoniae* Kp_Goe_152021, pKp_Goe_021-1, CP018714.1	5,373,055	58	246,756	45	5,979	48
7	*K. pneumoniae* Kp_Goe_827026, pKp_Goe_026-1, CP018708.1	5,373,056	57	246,756	45	6,034	48
8	*K. pneumoniae* Kp_Goe_827024, pKp_Goe_024-1, CP018702.1	5,374,118	57	246,753	45	6,034	48
9	*K. pneumoniae* Kp_Goe_149832, pKp_Goe_832-1, CP018696.1	5,373,057	57	246,755	45	6,034	48
10	*K. pneumoniae* MS6671.v1, LN824134.1	5,402,900	57	279,305	47	7,044	47
11	*K. pneumoniae*, pNDM-MAR, JN420336.1	*	*	267,242	47	8,215	46
12	*K. pneumoniae* A64477, pKP64477b, MF150122.1	*	*	205,089	45	6,282	47
13	*P. gergoviae* FB2, pFB2.1, CP014776.1	5,489,680	59	242,312	45	5,921	48
14	*K. pneumoniae* KPN528, pKPN528-1, CP020854.1	5,383,018	57	292,471	46	5,676	48
15	*K. pneumoniae* Kp_Goe_149473, pKp_Goe_473-1, CP018687.1	5,373,056	57	246,757	45	5,979	48
16	*K. pneumoniae* Kp_Goe_822579, pKp_Goe_579-1, CP018313.1	5,381,436	57	245,975	45	5,979	48
17	*K. pneumoniae* Kp_Goe_154414, pKp_Goe_414-1, CP018339.1	5,159,815	58	204,862	45	5,738	48
18	*K. pneumoniae* AR_0068, unitig_1, CP020068.1	5,357,430	57	276,460	47	5,678	48
19	*K. pneumoniae* 11, pIncHI1B_DHQP1300920, CP016921.1	5,184,828	58	283,369	46	5,678	48
20	*K. pneumoniae* KP617, KP-plasmid1, CP012754.1	5,416,282	57	273,628	46	5,678	48
21	*K. pneumoniae* PittNDM01, plasmid1, CP006799.1	5,348,284	58	283,371	46	5,678	48
22	*K. pneumoniae* SKGH01, unnamed 1, CP015501.1	5,490,611	57	281,190	47	7,036	49
23	*K. pneumoniae* PMK1, pPMK1-NDM, CP008933.1	5,317,001	57	304,526	47	8,521	46
24	*K. pneumoniae* KPNIH48, pKPN-edaa, CP026398.1	5,531,975	57	249,238	47	6,032	48
25	*K. pneumoniae* KPN1481, pKPN1481-1, CP020848.1	5,554,150	58	347,748	47	8,518	48
26	*K. pneumoniae* KSB2_1B, unnamed1, CP024507.1	5,228,889	58	310,025	47	5,678	48
27	*K. pneumoniae* KPNIH50, pKPN-bbef, CP026172.1	5,616,605	57	243,967	46	6,042	48
28	*K. pneumoniae* F44, p44-1, CP025462.1	5,460,465	57	261,706	48	5,434	48
29	*K. pneumoniae-53*, plasmid1, SGOL01000000	6,501,177	59	45,187	46	5,671	47
30	*K. pneumoniae-65*, plasmid 1, SGOK01000000	5,850,021	57	45,574	46	5,671	47
31	*K. pneumoniae-CR5*, plasmid 1, SGOJ01000000	5,871,238	59	125,699	43	6,284	48
A	*K. pneumoniae* K66-45, pK66-45-1, CP020902.1	5,380,605	57	338,512	48	6,078	46
B	*K. pneumoniae* AR_0158, tig00000727, CP021699.1	5,165,071	58	354,705	48	3,177	48
C	*K. pneumoniae* LS356, pKP8-2, CP025638.1	5,409,425	58	153,586	49	3,133	47
D	*K. oxytoca* pKOX3, p1, KY913897.1	*	*	239,374	47	1,085	48
	Average	5,422,844	57	251,035	46.2	5,875	47.7
	STD	286,043	0.0172	69,579	0.012	1,321	0.01

GC content comparison among the newly described Type IV-A CRISPR-Cas loci, the IncHI1B/IncFIB(Mar) plasmid and isolate chromosomal sequences of the host. *The strain chromosomal sequence was not available on GenBank; only the plasmid sequence was available.

### Characterization of the Type IV-A3 CRISPR-Cas System Found in *Enterobacteriaceae*

**FIGURE 2:**
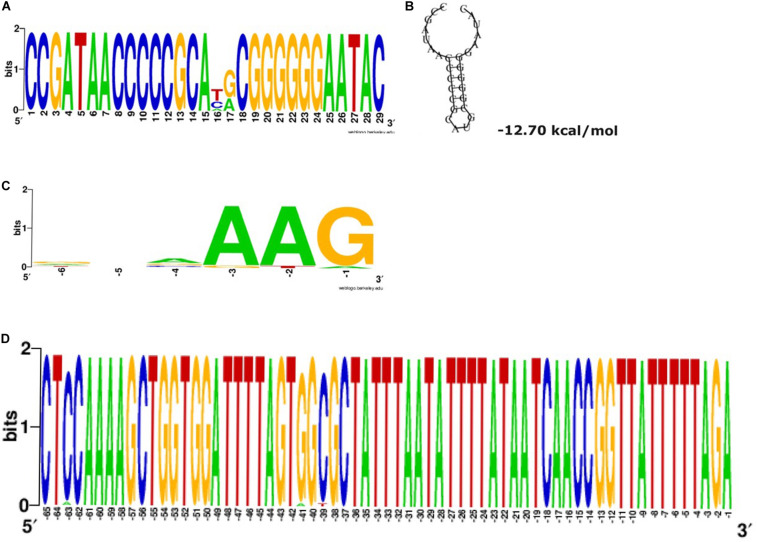
Type IV-A3 conserved repeats and the predicted stable stem-loop secondary structure, putative PAM and leader sequence. **(A)** Type IV-A-variant conserved repeats. The height of the letters in the sequence logo shows the relative frequency of their recurrence at that position. Wobbles at positions 16 and 17 are within the loop of the predicted stem-loop structure and are therefore tolerated in the structural prediction shown in **(B)**. **(B)** The predicted secondary structure of direct repeats and the associated Minimum Free Energy (MFE) estimated in (kcal/mol) shown underneath the structure. This structure is predicted to be involved in the mechanism of pre-crRNA processing. **(C)** Type IV-A-variant conserved putative protospacer adjacent motifs (PAMs). The alignment of the regions containing protospacers shows the conservation of putative PAM signature (AAG), position –3 to –1, adjacent to the end of the protospacers, using WebLogo. The analysis was performed on all the detected (467 spacers) of the 31 CRISPR1 arrays analyzed, specifically, spacer matching 9% (42/467) to bacteriophages and 25.5% (119/467) to plasmid sequences. Searches for other subtypes/variants were unsuccessful, likely due to the low number of spacer–protospacer matches **(D)** Conserved Type IV-A-variant CRISPR leader. The WebLogo shows a highly conserved 65 bp occurring between the CRISPR-Cas loci an the CRISPR array among the sequences investigated in this study.

A single CRISPR array (CRISPR1) was identified upstream of all *cas* loci. The repeats have a predicted stem-loop secondary-structure (Figures 2A,B) and is likely involved in a pre-crRNA Cas6-mediated process. The alignment of the regions around and containing the protospacer, particularly the last six positions preceding the protospacer, associated with CRISPR1 repeats revealed the conservation of the putative PAM signature (AAG) adjacent to the end of the protospacers (Figure 2C). A highly conserved 65 bp leader sequence occurring between the CRISPR-Cas loci and the CRISPR array was observed in all the sequences (Figure 2D). The minor variations in the leader sequence only occurred in two sequences (C in position −63 is A in CP014776.1 *Pluralibacter gergoviae*, and G in position −41 is A, and C in position −39 is T in *K. pneumoniae-CR5* ST-392). The high conservation of the leader sequence is unlike that presented in Pinilla-Redondo et al. (2019). RT-PCR of the confirmed *cas* loci (*cas7*, *dinG*, and *cas6*) demonstrated that they are expressed in all three of the available clinical isolates (Table 2).

**TABLE 2:** RT-PCR data of the confirmed *cas* loci (*cas7*, *dinG*, and *cas6*) in the three clinical isolates.

Isolate	Gene^*‡*^			
	rpoB*	*cas7/csf2*	*dinG/csf4*	*cas6/csf5*
***K. pneumoniae-CR5***	16.71	22.385	23.865	23.69
***K. pneumoniae-CR5*** RT-ve CTRL**	31.21	37.48	42.52	34.755
***K. pneumoniae-53***	16.595	24.2	25.305	25.325
***K. pneumoniae-53*** RT-ve CTRL**	32.65	37.74	34.37	33.61
***K. pneumoniae-65***	17.575	23.2	24.745	24.44
***K. pneumoniae-65*** RT-ve CTRL**	32.23	42.79	46	34.045

^†^Genes amplification data represent the average of two experiments at least. The cut-off cycle threshold (Ct) was 30 cycles. *rpoB housekeeping gene was used as the internal positive control. **RT-ve CTRL represent no addition of the reverse transcriptase which was used as the negative control for residual DNA.

**FIGURE 3:**
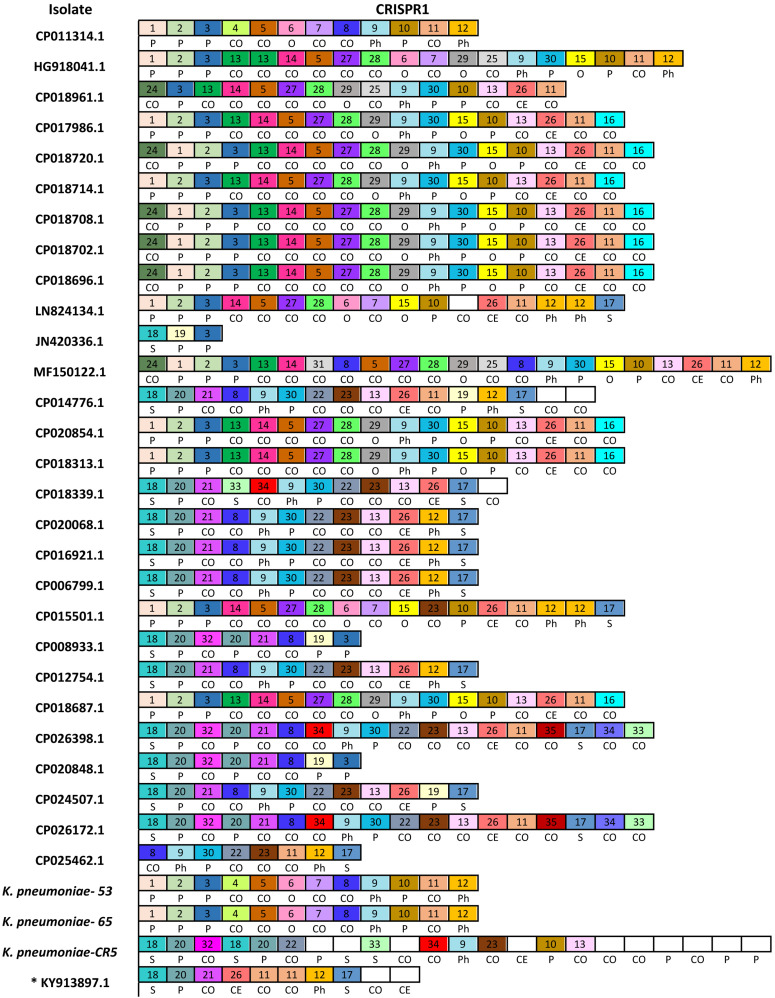
Newly described Type IV-A3 CRISPR spacer polymorphism. The spacers map. Only spacers are represented by boxes, and no repeats are included. Identical spacers are represented by the same number and color, while unique spacers are represented by white color and no number is associated with the box. Self-targeting spacers are indicated by letter (S) and show 100% identity to host DNA, plasmid-targeting spacers are indicated by letter (P), phage targeting spacers are indicated by letters (Ph), other *Enterobacteriaceae* targeting spacers (100% identity) are indicated by letter (O), cryptic spacers with similarity to other bacterial DNA are indicated by letters (CO), and those with similarity to Eukaryotic DNA are indicated by letters (CE) that are positioned underneath the relevant spacer. CE spacers showed at least 57% identity to eukaryotic DNA. CE spacers were confirmed by multiple sequences alignments. * KY913897.1 is the isolate that only has a CRISPR array and a *cas7* (*csf2*) (not a complete Type IV system) and therefore is not included in the total analysis in Figure 4.

**FIGURE 4:**
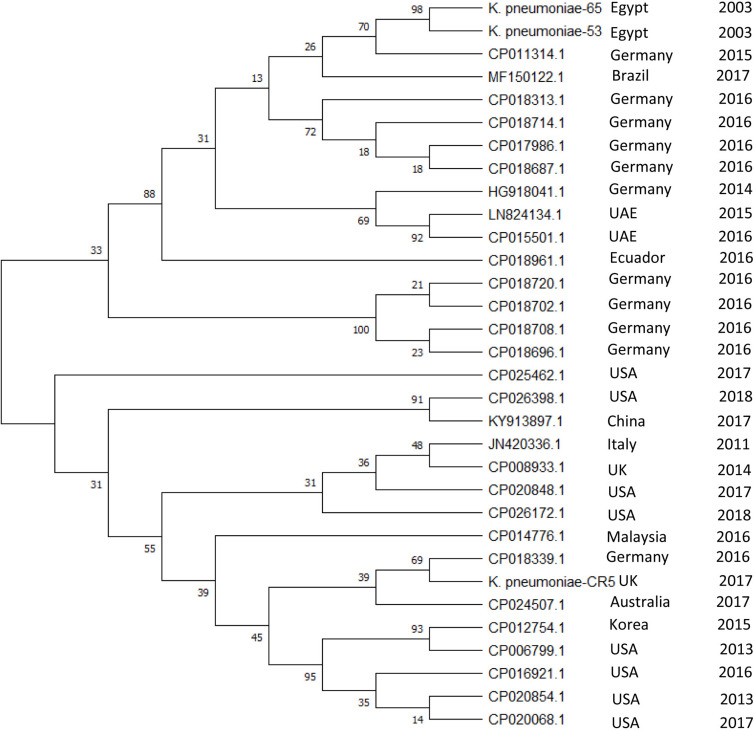
Evolutionary relationships of Type IV-A3 CRISPR spacer. The phylogenetic tree illustrating the evolutionary relationships of Type IV-A3 CRISPR array nucleotide sequences. Phylogenetic UPGMA tree was constructed using the MUSCLE algorithm of MEGA7. The evolutionary distances were computed using the Maximum Likelihood method and Tamura-Nei model, bootstrap test (1000 replicates), and the rate variation among sites was modeled with a gamma distribution (shape parameter = 2). The percentage of trees in which the associated taxa clustered together is shown next to the branches. The year and geographical origin of the isolate are listed to the right of the branch ends.

We have detected a total of 467 spacers in the 31 CRISPR1 arrays analyzed, out of which 9% (42/467) match to bacteriophages and 25.5% (119/467) match to plasmid sequences. The majority of spacer sequences are present in more than one spacer array and some are present more than once within the same array (Figure 3). Plasmid targeting spacers appeared in every example of this Type IV-A3 associated CRISPR array analyzed. Sequence analysis revealed that spacers correspond to *IncFIIK* conjugal transfer genes; *traN* and *traL* (Figure 3). Limited conservation within the order of the spacer arrays showed that the arrays cluster into two distinct groups which share geographical associations and suggest persistence within the plasmid pool in isolates from certain countries over time (Figure 4).

The CRISPR system described here is always found associated with *IncH1B*/*IncFIB* plasmids in *Enterobacteriaceae*, has *dinG* and *cas7* (involved in interference), and *cas6, cas5, and cas8-like* (involved in expression and maturation of short crRNAs) (Cass et al., 2015; Dwarakanath et al., 2015; Koonin and Krupovic, 2015; Lundgren et al., 2015; Crowley et al., 2019; Faure et al., 2019b). The detection of a *csf1*/*cas8-like* in the Type IV-A-variant described here updates the initial (Newire et al., 2019) and subsequent (Kamruzzaman and Iredell, 2019) reports of this system. Additionally, our results agree with other reports suggesting a need for Type IV-A variant classification (Crowley et al., 2019; Pinilla-Redondo et al., 2019).

Notably, some of the previously described Type IV systems do not possess a *dinG* or *cas8-like* (e.g., Type IV-C); however, *cas7* genes are consistently found in all the previously and presently described Type IV sequences. Also, Cas7 is the most conserved protein among members of the Type IV CRISPR family (Pinilla-Redondo et al., 2019). This highlights the role of *cas7* in Type IV identification.

This Type IV-A3 described here has a variable CRISPR array and a conserved leader sequence. The conserved leader sequence occurrence in a wide variety of *K. pneumoniae* sequence types may reflect their narrow association with *IncH1B*/*IncFIB* plasmids. Conserved leader sequences in other types (Type I-E) were shown to increase acquisition efficiency by presumably stabilizing the Cas1–2-leader-repeat interaction (Kieper et al., 2019). The order of spacers demonstrated conservation with some polymorphism, and they cluster into two main groups (Figure 4) matching DNA from a variety of geographical sources. Expression of this Type IV-A *cas* genes suggests immunity to incoming DNA matching the spacers. Crowley et al. (2019) posit that interference is mediated, similar to type I and type III systems, through multi-subunit complexes composed of Csf proteins and the use of crRNA as a guide to bind complementary nucleic acid forming R-loops. In this case, it was hypothesized that DinG is then recruited to these R-loops, where it either acts directly to destroy the foreign DNA (e.g., a plasmid) or recruits an endogenous nuclease to mediate RNA-guided interference (Crowley et al., 2019); however, this needs to be tested. Also, we note that the adaptation module is missing, thus adding new spacers will require *cas1* and *cas2* from other CRISPR-Cas systems. Like other Type IV systems that cannot function as independent adaptive immune systems (Koonin and Krupovic, 2015), we suspect that the Type IV-A3 CRISPR-Cas described here is likely to co-operate with other *cas* loci, whenever they exist within the *Enterobacteriaceae* host genomes, for spacer acquisition. Those CRISPR-Cas loci could belong to those CRISPR systems that are known to be frequently associated with an *Enterobacteriaceae* host, such as Type I-E/I-E^∗^ or Type I-F (Aydin et al., 2017). The association between Type IV-A and *cas6e* and *cas6f* (*cas6* sequences observed in subtypes I-E and I-F, respectively) was previously reported in other bacterial families, suggesting functional links (Pinilla-Redondo et al., 2019; Taylor et al., 2019). These functional links were inferred based on the similarities in the leader and the repeat sequences of Type I-E and Type IV-A3 (Pinilla-Redondo et al., 2019). Another evidence that supports possible functional co-operation is the presence of Cas6 that shows 99%+ identity to Type I-E Cas6 in *Enterobacteriaceae* in the Type IV-A3 described here. Furthermore, unlike other Cas proteins associated with Type IV-A3 described here, Cas6 were highly conserved sequences, showing no particular association with interrupting IS elements, which may further support the recruitment of *cas6* is form Type I-E. These possible adaptation functional links appear to be a feature that can be switched on/off, which requires the presence of the *IncHI1B/IncFIB(Mar)* plasmids (that carry this Type IV-A-variant) inside a bacterial host that has a functional CRISPR-Cas system in its genome. Although a previous report suggested that Type IV CRISPR-Cas system-positive plasmids were only found in *Enterobacteriaceae* with chromosomal Type I-E/I-E^∗^ CRISPR-Cas (Kamruzzaman and Iredell, 2019), we could not identify Type I-E/I-E^∗^ in all the isolate genomes that have Type IV-A3. For example, *K. pneumoniae-53*, CP011314.1, HG918041.1, JN420336.1, MF150122.1, CP014776.1, CP018339.1, CP026398.1, CP020848.1, CP024507.1, CP026172.1, and CP025462.1 did not have Type I-E/I-E^∗^ CRISPR-Cas systems. Thus, we assume there is no conditional connection between the presence of Type IV-A3 and Type I-E/I-E^∗^ CRISPR-Cas systems in *Enterobacteriaceae*.

Type IV-A3 CRISPR system reported here is exclusively located on *IncH1B*/*IncFIB* plasmids. We have also spotted an imperfect spacer target in *traN* of an *IncFIIK* plasmid in *K. pneumoniae-53* which suggests this plasmid may be able to evade plasmid mediated CRISPR interaction within this strain (Jiang et al., 2013). Therefore, these spacers are likely to be involved in plasmid competition; protecting the resident Type IV-A CRISPR-Cas carrying plasmid in *Enterobacteriaceae* as previously suggested (Newire et al., 2019; Pinilla-Redondo et al., 2019). Recently, some Type IV-A system variants that are associated with *P. aeruginosa* were shown to target invasive plasmids, which strengthens the involvement of Type IV-A CRISPR-Cas systems in plasmid competition (Crowley et al., 2019).

Type IV CRISPR-Cas systems demonstrate a notable diversity of molecular organization (Figure 1) and some appear to have taken on roles in addition to adaptive cellular immunity (Faure et al., 2019a). For example, some of the Type IV CRISPR–Cas loci were previously predicted to encode bacterial toxins that together with the Cas proteins of the Type IV systems may contribute to plasmid stabilization (Faure et al., 2019b). The Type IV-A3 system described here demonstrates a complex evolutionary connection with MGEs in terms of parasitism and immunity (Koonin and Makarova, 2017). The association between this Type IV-A3 system and multiple MGEs, plus the identification of partial *cas* loci genes with and without the CRISPR array on other *IncHI1B/IncFIB(Mar)* plasmids (Figure 1 and Supplementary Table S1), plus the identification of similar arrays in different plasmids in the same host from the same country (Figures 3, 4), indicates that dynamic, MGE mediated movement and rearrangement of this CRISPR-Cas Type IV-A system is ongoing. The similarity in the GC content between this Type IV-A and the *IncHI1B/IncFIB* plasmids in contrast with the higher chromosomal GC content supports the observations that the system is exclusively plasmid associated, both in this study and in others (Ravenhall et al., 2015). Because reporting standard deviations from comparisons of element-wide GC contents across different genomes could be misleading, since the strains are closely related that statistical observations are not independent, we have investigated the GC skew in a reasonably sized sliding window (1000 bp) across the length of the element (Type IV-A3 system and plasmid DNA sequences) in a single genome (*K. pneumoniae-65*), which also confirmed that the system is exclusively plasmid associated. This demonstrates unique evolutionarily juxtaposed connections between CRISPR-Cas and MGEs which is worthy of further investigation. To our knowledge, this is the first identification of a CRISPR-Cas system exclusively associated with *IncHI1B/IncFIB* plasmids that demonstrates an evolutionary association with MGEs and is likely to be involved in plasmid competition.

## Author’s Note

This manuscript has been released as a pre-print at BioRxiv (Newire et al., 2019).

## Data Availability Statement

The datasets presented in this study can be found in online repositories. The names of the repository/repositories and accession number(s) can be found at: https://www.ncbi.nlm.nih.gov/genbank/, SGOJ00000000; https://www.ncbi.nlm.nih.gov/genbank/, SGOK00000000; and https://www.ncbi.nlm.nih.gov/genbank/, SGOL00000000.

## Author Contributions

EN discovered the CRISPR system within the genomes of her Egyptian isolate collection, analyzed the sequence data, and wrote the first draft of the manuscript. SJ, AA, and VE designed and carried out the experiments to test *cas* loci expression. AR analyzed the data and wrote the manuscript. All authors critically reviewed and approved the manuscript.

## Conflict of Interest

The authors declare that the research was conducted in the absence of any commercial or financial relationships that could be construed as a potential conflict of interest.

## Footnotes

1 https://toolkit.tuebingen.mpg.de/tools/hhpred

2 http://genskew.csb.univie.ac.at/

3 https://blast.ncbi.nlm.nih.gov/
