# Supplementary material for: Identification of a Type IV-A CRISPR-Cas System Located Exclusively on IncHI1B/IncFIB Plasmids in Enterobacteriaceae
Source: Front Microbiol. 2020 Aug 12;11:1937. doi: 10.3389/fmicb.2020.01937 (PMC7434947; doi:10.3389/fmicb.2020.01937)
Supplement: Supplementary file 1 [file Table_1.docx]

**Supplementary Table S1: Type IV-A-variant *IncHI1B/ IncFIB (Mar)* plasmid information. †**

|  | **Strain**  **(Accession number)** | **ST** | **location (date)** | **plasmid** | **Resistance genes** | **Repeat-spacer CRISPR loci*** | | | | | **Novel Type IV Structure** |
| --- | --- | --- | --- | --- | --- | --- | --- | --- | --- | --- | --- |
|  |  |  |  |  |  | **Start** | **End** | **Spacer number** | **Length of DR** | **CRISPR length** |  |
| 1 | *K. pneumoniae* strain 234-12 plasmid pKpn23412-362 (CP011314.1) | ST-514 | Germany (2015) | *IncHI1B* | *merC, aph(6)-Id, tmrB, terY, terX, terW, terZ, terA, terB, terC, bla*_TEM-1_*, bla*_CTX-M-15,_ *bla*_OXA-1_*, aacA4, aph(3''), aac(3)-IIa, aac(6')Ib-cr, strA, strB, catB4, catA1, sul2, tet(A), dfrA1* | 141865 | 142618 | 12 | 23 | 753 | 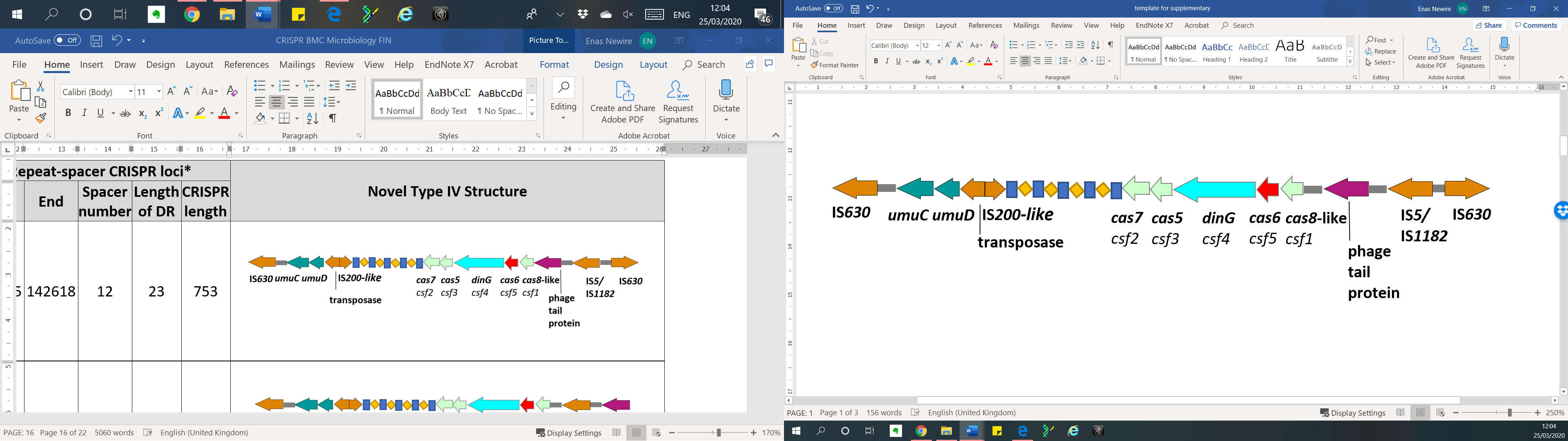 |
| 2 | *K. pneumoniae* Kp15 plasmid pENVA (HG918041.1) | unknown | Germany  (2014) | *IncH* | *merD, merA, merP, qnrb4, aadA, terY, terX, terW, terZ, terA, terB, terC, bla*_CTX-M-15_*, bla*_DHA-1_*, qacE, bla*_TEM-1_*, aadA1, aac(3)-II, sul1, tet(A), dfrA15* | 99454 | 100629 | 19 | 23 | 1175 | 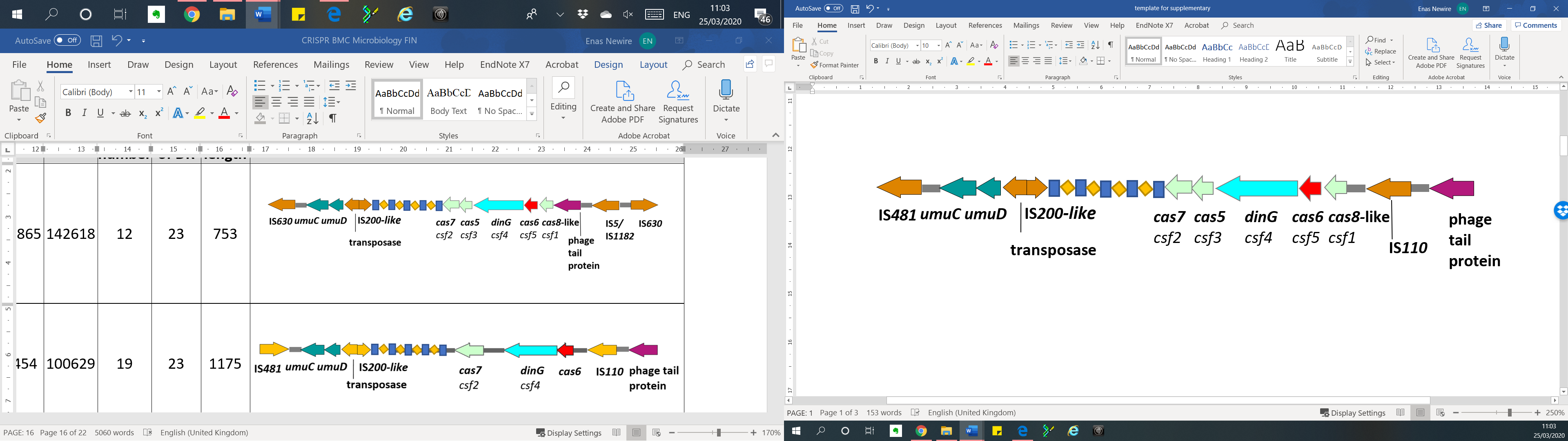 |
| 3 | *E. coli* strain Ecol_422 plasmid pEC422_1 (CP018961.1) | ST-2 | Ecuador (2016) | *IncHI1B* | *tetC, sul1, mer, qnrE1, terY, terX, terW, terZ, terA, terB, terC, bla*_CTX-M-2_*, qacE, bla*_OXA-1_*, bla*_TEM-26_*, aac(6')Ib-cr, aac(3)-IIa, mph(A), catB3,arr-3, sul1* | 276922 | 277868 | 15 | 29 | 946 | 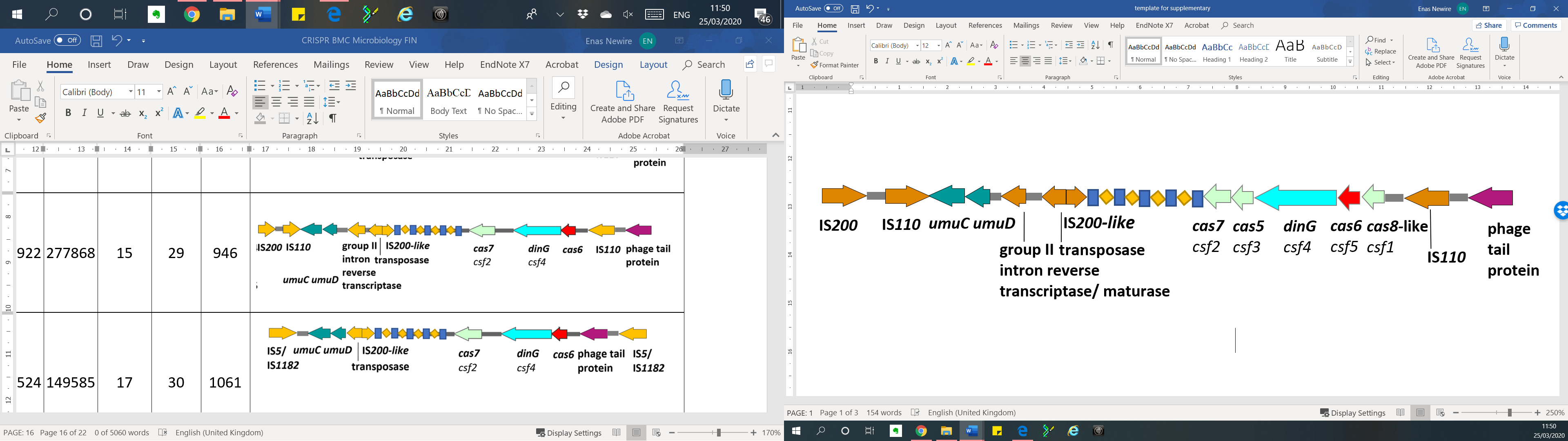 |
| 4 | *K. pneumoniae* strain 825795-1 plasmid unnamed1 (CP017986.1) | ST-147 | Germany  (2016) | *IncHI1B* | *terA* | 148524 | 149585 | 17 | 30 | 1061 | 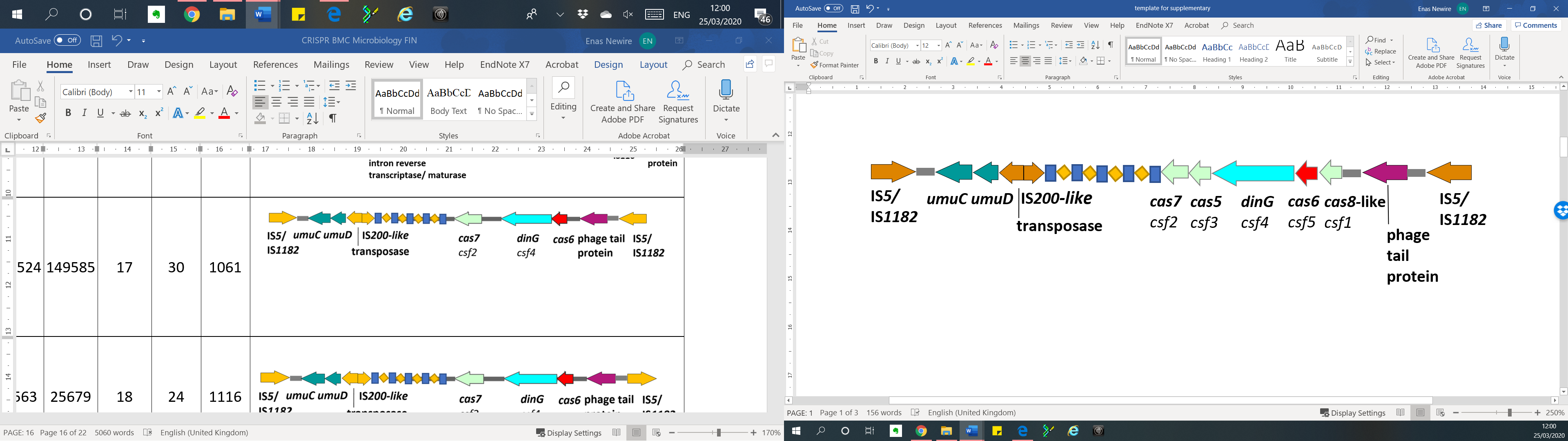 |
| 5 | *K. pneumoniae* strain KP_Goe_828304 plasmid pKp_Goe_304-1 ([CP018720.1](https://www.ncbi.nlm.nih.gov/nucleotide/1120823956?report=genbank&log$=nuclalign&blast_rank=6&RID=PTB95N0Y01R)) | ST-147 | Germany  (2016) | *IncHI1B* | *terY, terX, terW, terZ, terA, terB, terC* | 24563 | 25679 | 18 | 24 | 1116 | 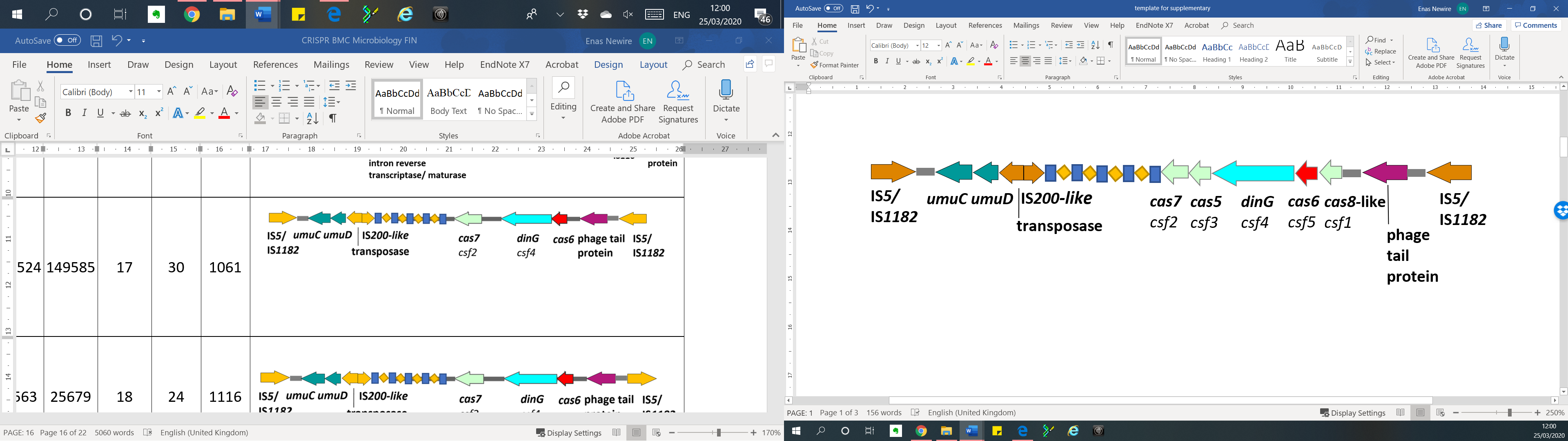 |
| 6 | *K. pneumoniae* strain Kp_Goe_152021 plasmid pKp_Goe_021-1  (CP018714.1) | ST-147 | Germany  (2016) | *IncHI1B* | *terY, terX, terW, terZ, terA, terB, terC* | 7687 | 8748 | 17 | 30 | 1061 | 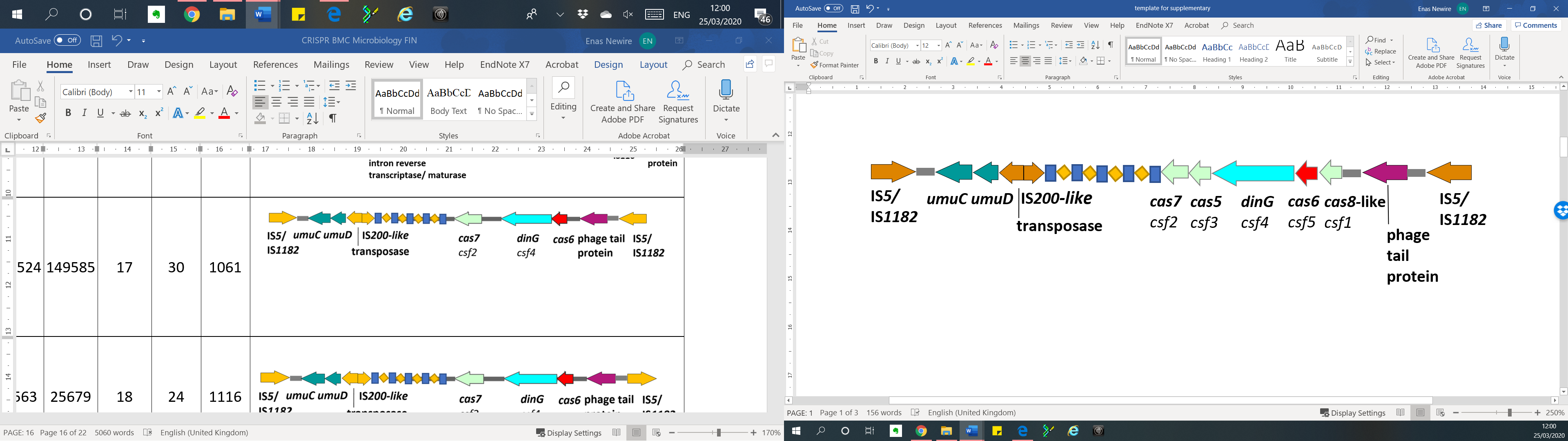 |
| 7 | *K. pneumoniae* strain Kp_Goe_827026 plasmid pKp_Goe_026-1 (CP018708.1) | ST-147 | Germany  (2016) | *IncHI1B* | none | 54661 | 55777 | 18 | 24 | 1116 | 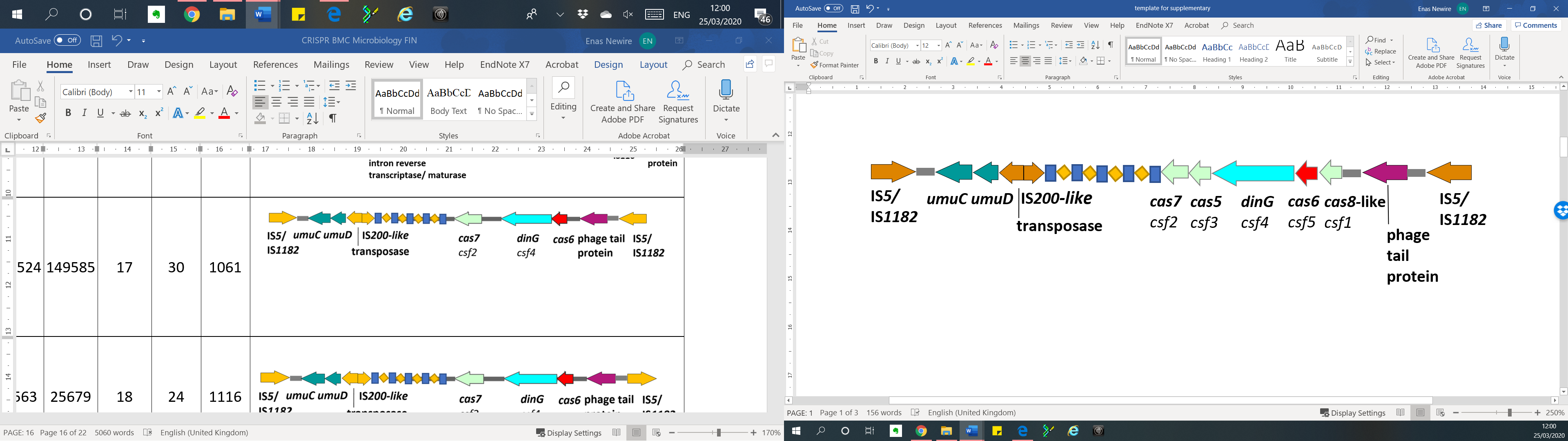 |
| 8 | *K. pneumoniae* strain Kp_Goe_827024 plasmid pKp_Goe_024-1 (CP018702.1) | ST-147 | Germany  (2016) | *IncHI1B* | *terY, terX, terW, terZ, terZ, terB, terC* | 5006 | 6122 | 18 | 24 | 1116 | 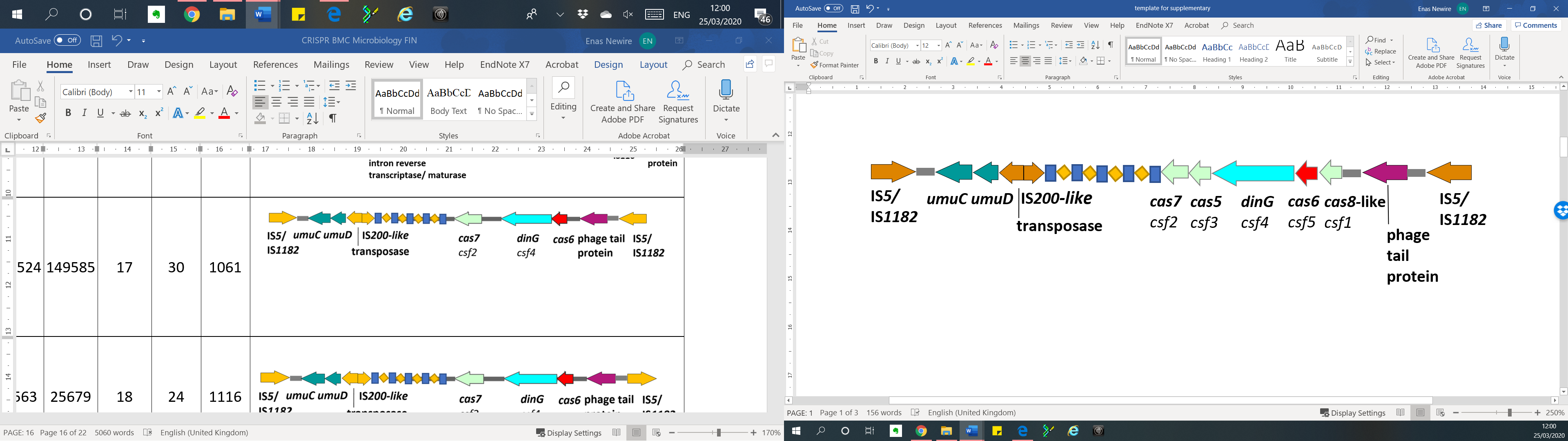 |
| 9 | *K. pneumoniae* strain Kp_Goe_149832 plasmid pKp_Goe_832-1  (CP018696.1) | ST-147 | Germany  (2016) | *IncHI1B* | *terY, terX, terW, terZ, terA, terB, terC* | 102143 | 103259 | 18 | 24 | 1116 | 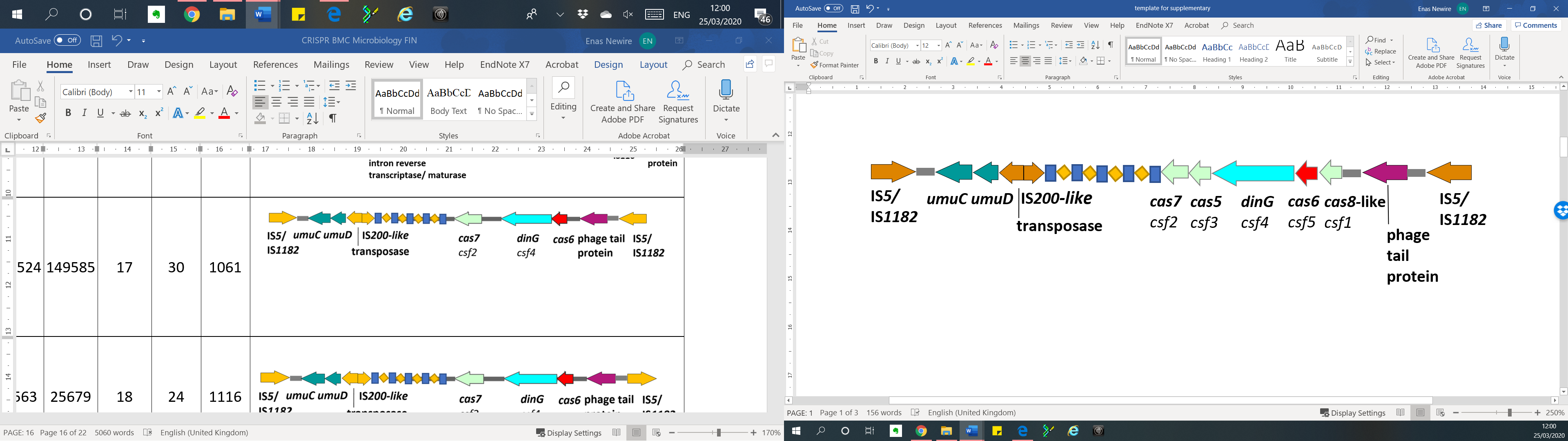 |
| 10 | *K. pneumoniae* MS6671.v1, plasmid (LN824134.1) | ST-147 | UAE  (2015) | *IncHI1B* | none | 16764 | 17823 | 17 | 29 | 1059 | 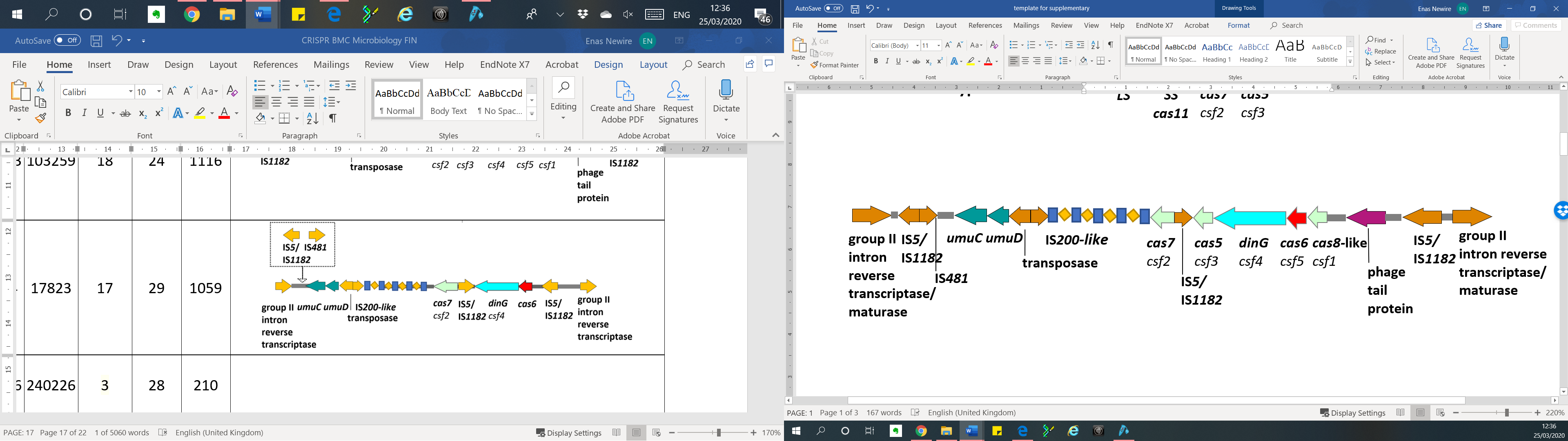 |
| 11 | *K. pneumoniae* plasmid pNDM-MAR (JN420336.1) | ST-15 | Italy (2011) | IncH | *bla*_NDM-1_*, ble*_MBL_*, qnrB66, merR, terY3, terY1, terW, terZ, terA, terC, terD, terE, terF, bla*_CTX-M-15_*, bla*_OXA-1_*, aac(6')Ib-cr, aac(6')Ib-cr, catA1, catB4* | 240016 | 240226 | 3 | 28 | 210 | 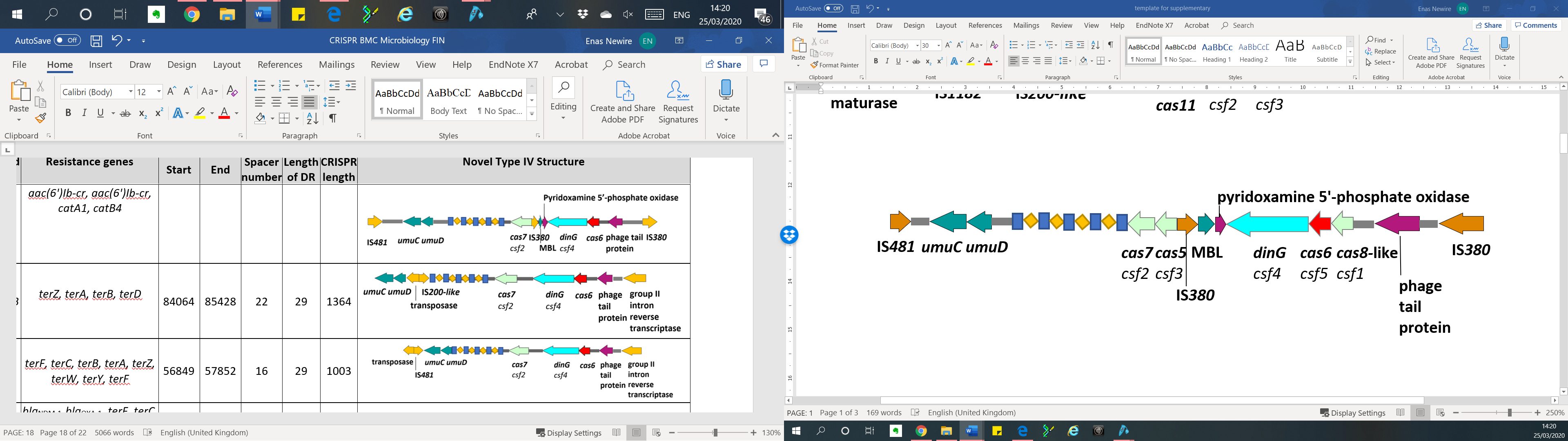 |
| 12 | *K. pneumoniae* A64477 plasmid pKP64477b (MF150122.1) |  | Brazil (2017) | *IncHI1B* | *terZ, terA, terB, terD* | 84064 | 85428 | 22 | 29 | 1364 | 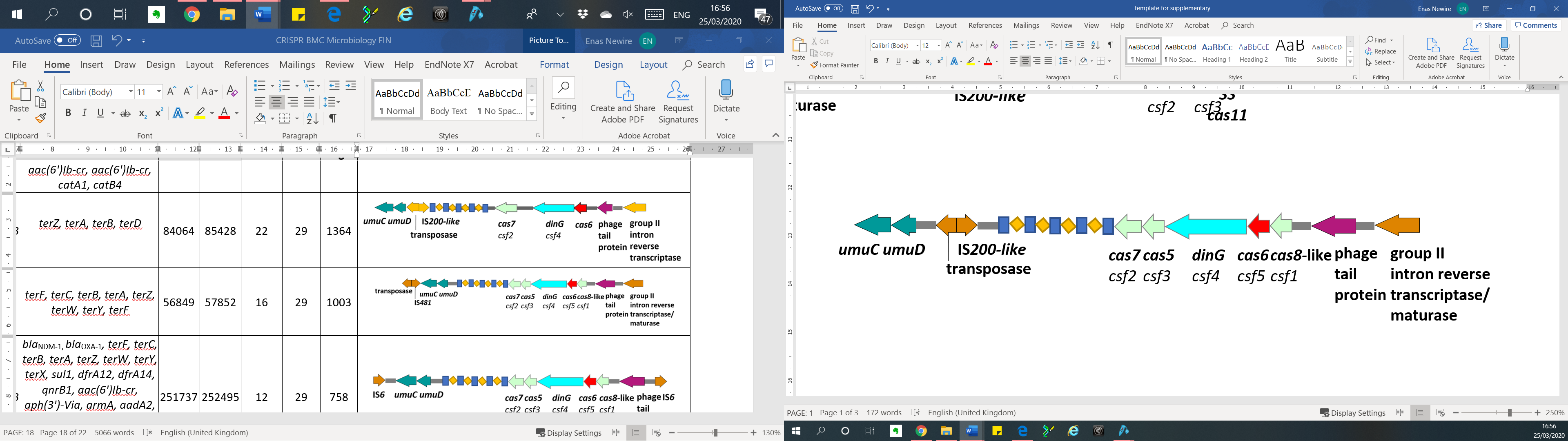 |
| 13 | *P. gergoviae* FB2 plasmid pFB2.1 (CP014776.1) | unknown | Malaysia (2016) | *IncFIB (Mar)* | *terF, terC, terB, terA, terZ, terW, terY, terF* | 56849 | 57852 | 16 | 29 | 1003 | 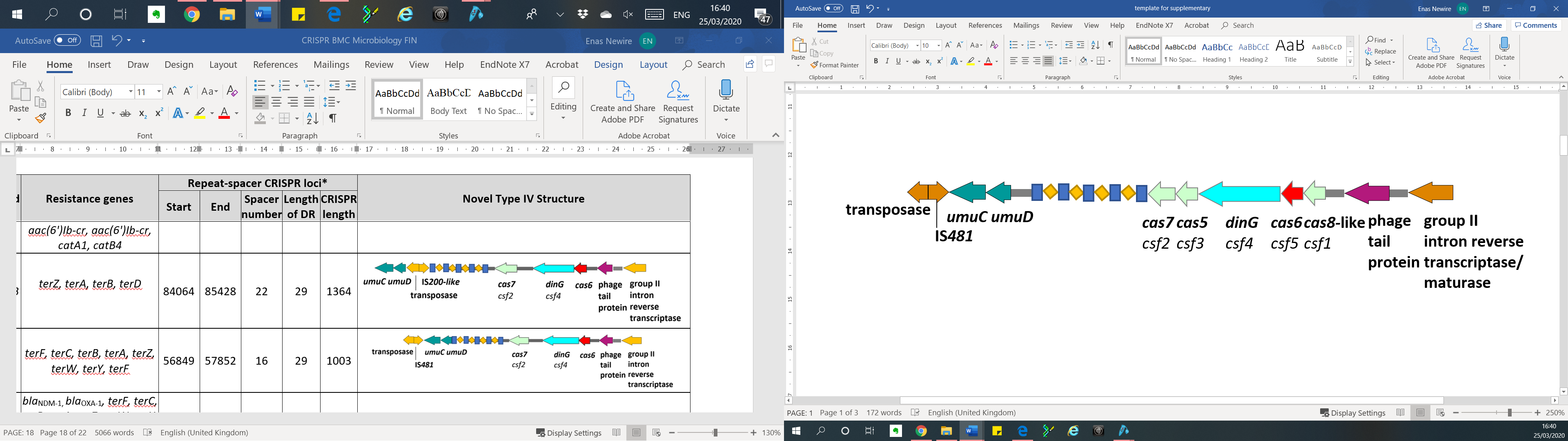 |
| 14 | *K. pneumoniae* KPN528 plasmid pKPN528-1  (CP020854.1) | ST-14 | USA (2013) | *IncHI1B* | *bla*_NDM-1,_ *bla*_OXA-1_*, terF, terC, terB, terA, terZ, terW, terY, terX, sul1, dfrA12, dfrA14, qnrB1, aac(6')Ib-cr, aph(3')-Via, armA, aadA2, aac(6')Ib-cr, mph(E), msr(E), catB4* | 251737 | 252495 | 12 | 29 | 758 | 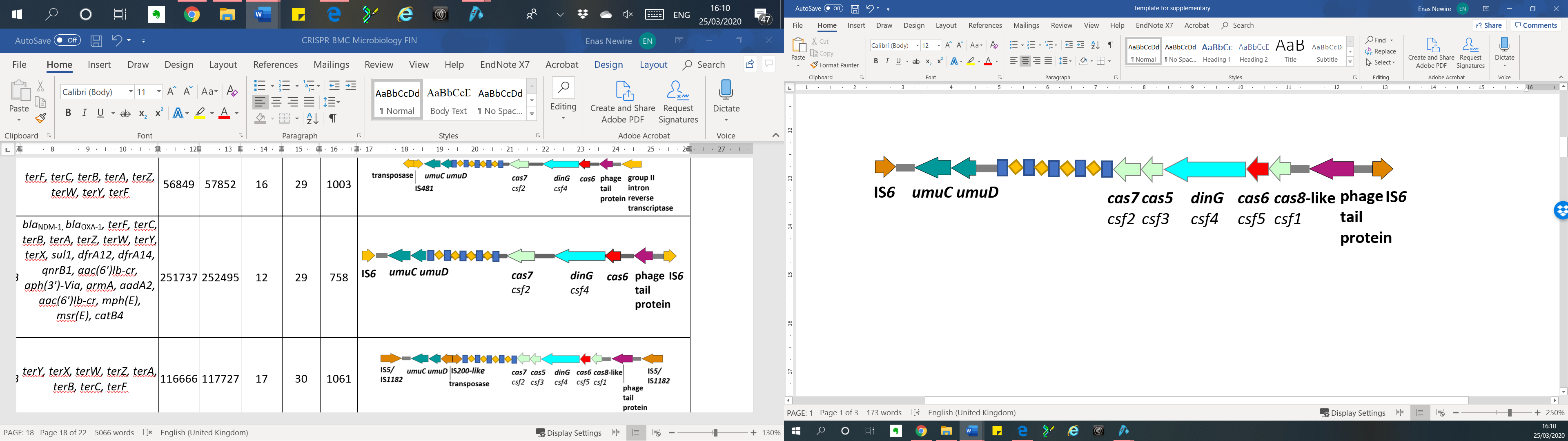 |
| 15 | *K. pneumoniae* Kp_Goe_149473 plasmid pKp_Goe_473-1 (CP018687.1) | ST-147 | Germany  (2016) | *IncHI1B* | *terY, terX, terW, terZ, terA, terB, terC, terF* | 116666 | 117727 | 17 | 30 | 1061 | 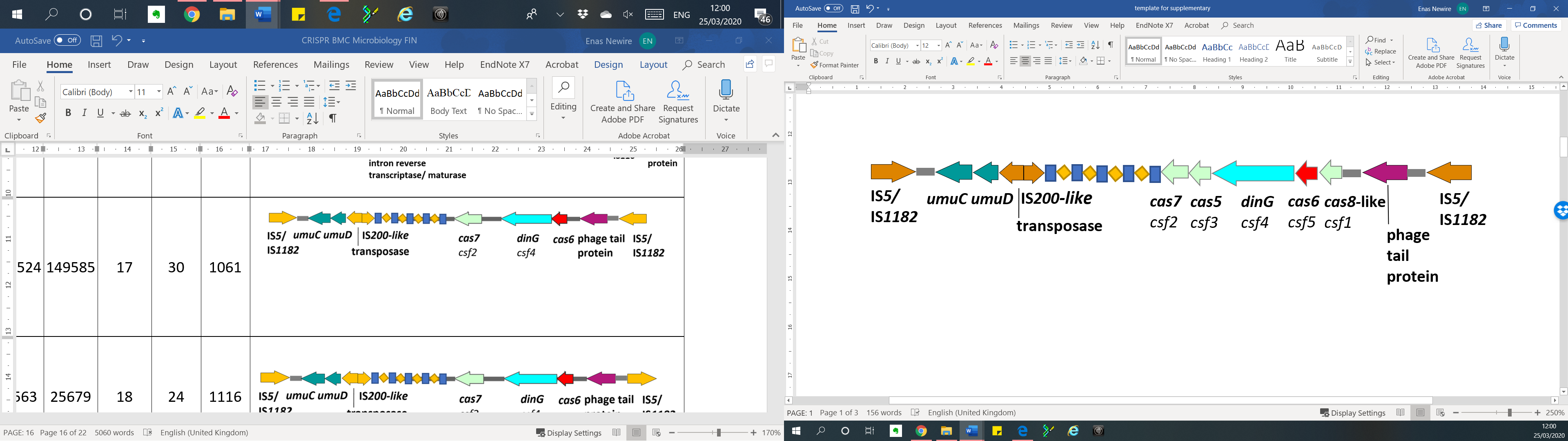 |
| 16 | *K. pneumoniae* strain Kp_Goe_822579 plasmid pKp_Goe_579-1 (CP018313.1) | ST-147 | Germany  (2016) | *IncHI1B* | none | 94773 | 95834 | 17 | 30 | 1061 | 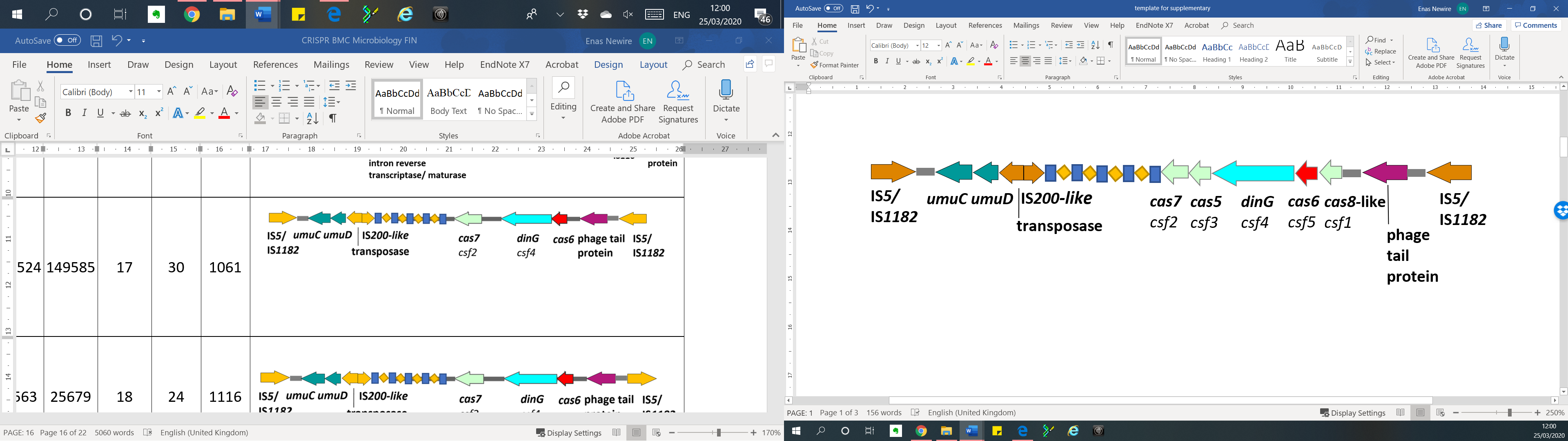 |
| 17 | *K. pneumoniae* Kp_Goe_154414 plasmid pKp_Goe_414-1 (CP018339.1) | ST-23 | Germany  (2016) | *IncFIB (Mar)* | *terY, terX, terW, terZ, terA, terB, terC, terF* | 75291 | 76111 | 13 | 29 | 820 | 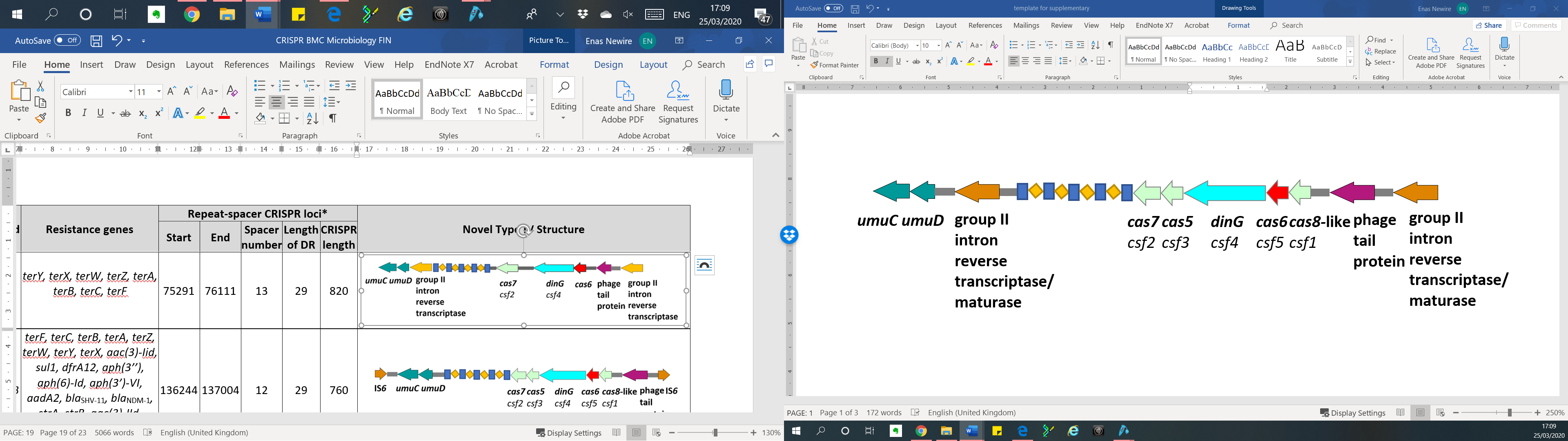 |
| 18 | *K. pneumoniae* AR_0068 plasmid unitig_1 (CP020068.1) | ST-14 | USA  (2017) | *IncHI1B* | *terF, terC, terB, terA, terZ, terW, terY, terX, aac(3)-Iid, sul1, dfrA12, aph(3’’), aph(6)-Id, aph(3’)-VI, aadA2, bla*_SHV-11_*, bla*_NDM-1_*, strA, strB, aac(3)-IId, armA, aadA2, mph(E), msr(E), sul2* | 136244 | 137004 | 12 | 29 | 760 | 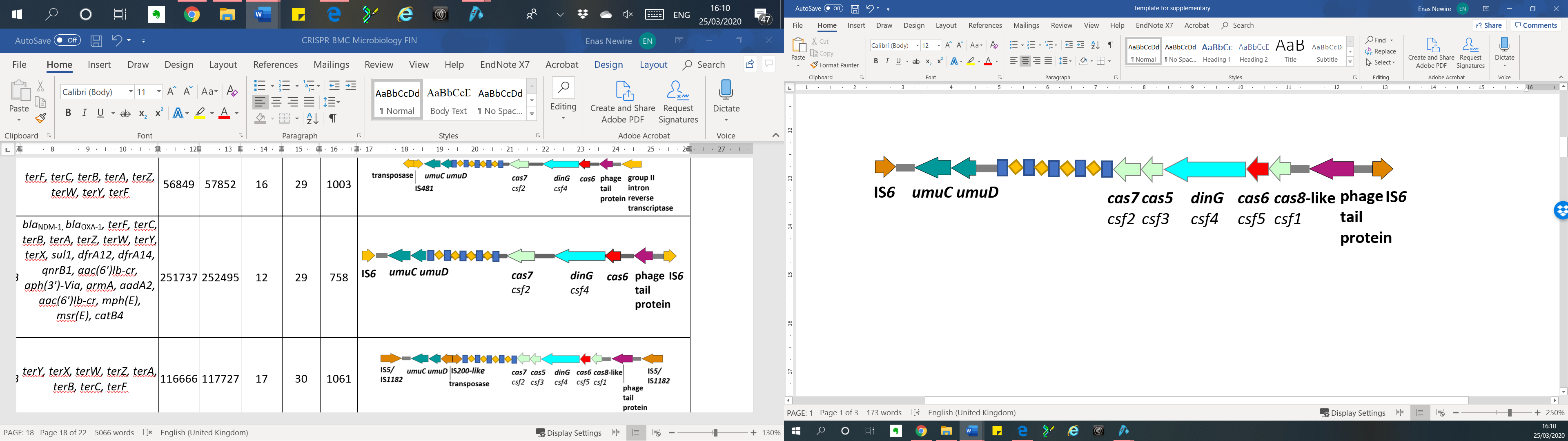 |
| 19 | *K. pneumoniae* 11 plasmid pIncHI1B_DHQP1300920 ([CP016921.1](https://www.ncbi.nlm.nih.gov/nucleotide/1057968381?report=genbank&log$=nuclalign&blast_rank=16&RID=P6PVYKC5015)) | ST-14 | USA  (2016) | IncHI1B | *terZ, terA, terB, terC, terF, dfrA1, terY, terX, terW, aph(3’), aacA4, ant(3’’), bla*_NDM-1_*, bla*_OXA-1_*, aac(6')Ib-cr, armA, aadA2, aac(6')Ib-cr, qnrB1, mph(E), msr(E), catB4, sul, dfrA14, dfrA12* | 229597 | 230357 | 12 | 29 | 760 | 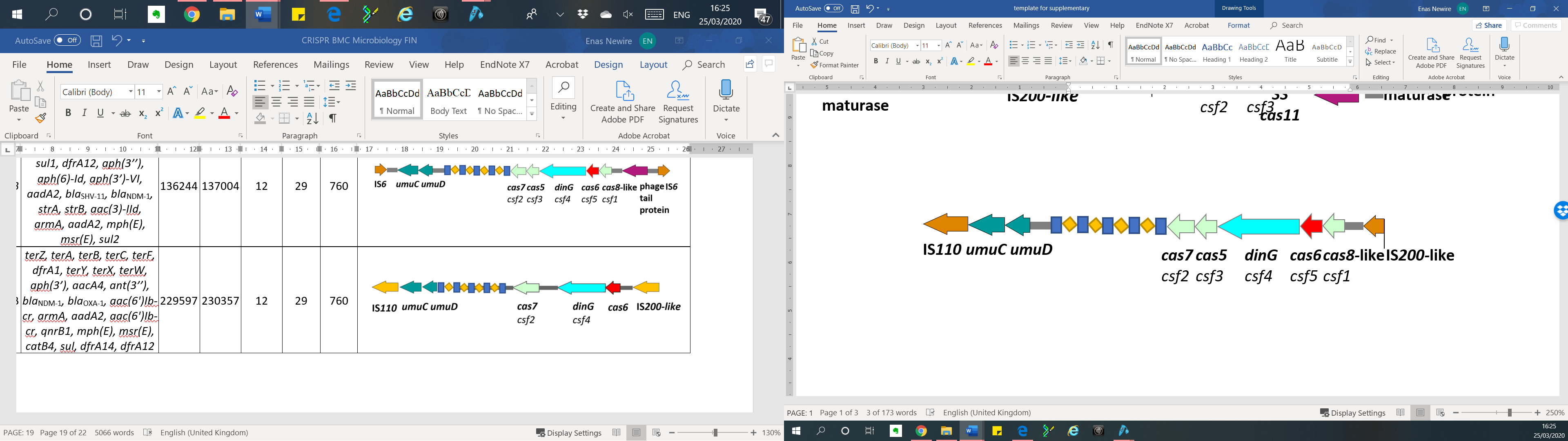 |
| 20 | *K. pneumoniae* KP617 plasmid KP-plasmid1  ([CP012754.1](https://www.ncbi.nlm.nih.gov/nucleotide/937297641?report=genbank&log$=nuclalign&blast_rank=17&RID=P6PVYKC5015)) | ST-14 | Korea  (2015) | *IncHI1B* | *terY, terX, terW, terZ, terA, terB, terC, terF, qnrB1, qacEDelta1, ble*_MBL_*, merE, aph(3’)-VI, bla*_NDM-1_  *aadA2, armA, msr(E), mph(E), sul1, dfrA12* | 114404 | 115164 | 12 | 29 | 760 | 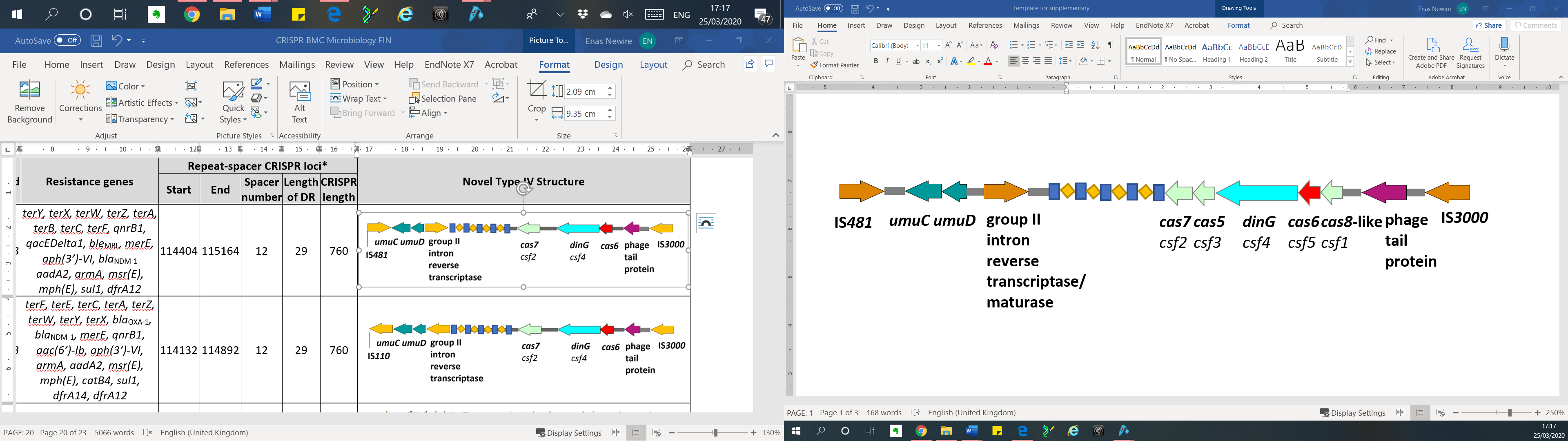 |
| 21 | *K. pneumoniae* PittNDM01 plasmid1 ([CP006799.1](https://www.ncbi.nlm.nih.gov/nucleotide/667713100?report=genbank&log$=nuclalign&blast_rank=18&RID=P6PVYKC5015)) | ST-14 | USA  (2013) | *IncHI1B* | *terF, terE, terC, terA, terZ, terW, terY, terX, bla*_OXA-1_*, bla*_NDM-1_*, merE, qnrB1, aac(6’)-Ib, aph(3’)-VI, armA, aadA2, msr(E), mph(E), catB4, sul1, dfrA14, dfrA12* | 114132 | 114892 | 12 | 29 | 760 | 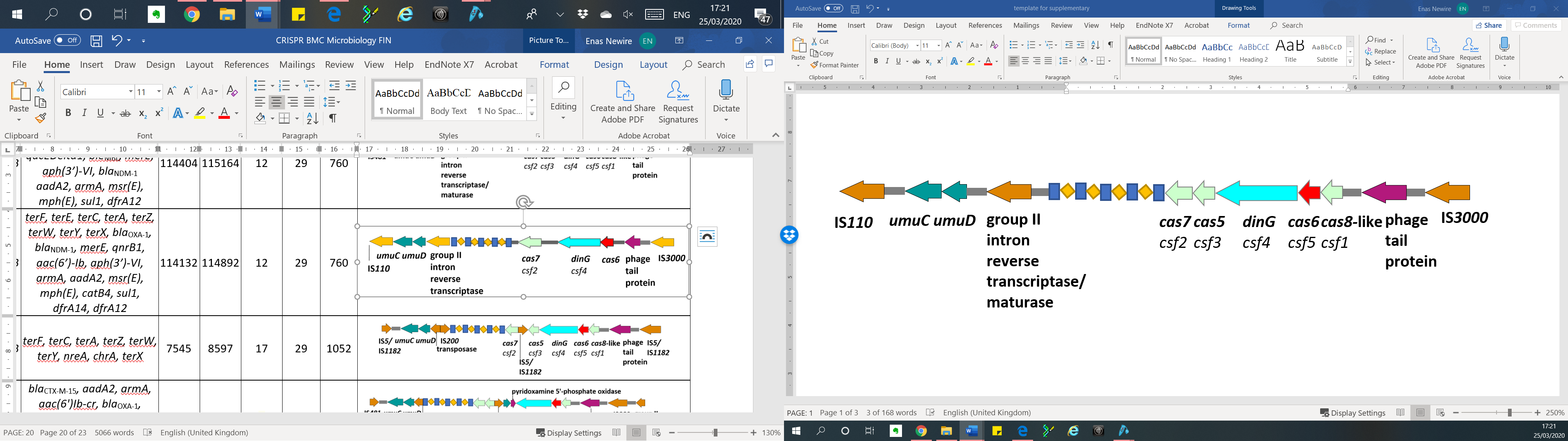 |
| 22 | *K. pneumoniae* SKGH01 plasmid unnamed 1 (CP015501.1) | ST-147 | UAE  (2016) | *IncHI1B* | *terF, terC, terA, terZ, terW, terY, nreA, chrA, terX* | 7545 | 8597 | 17 | 29 | 1052 | 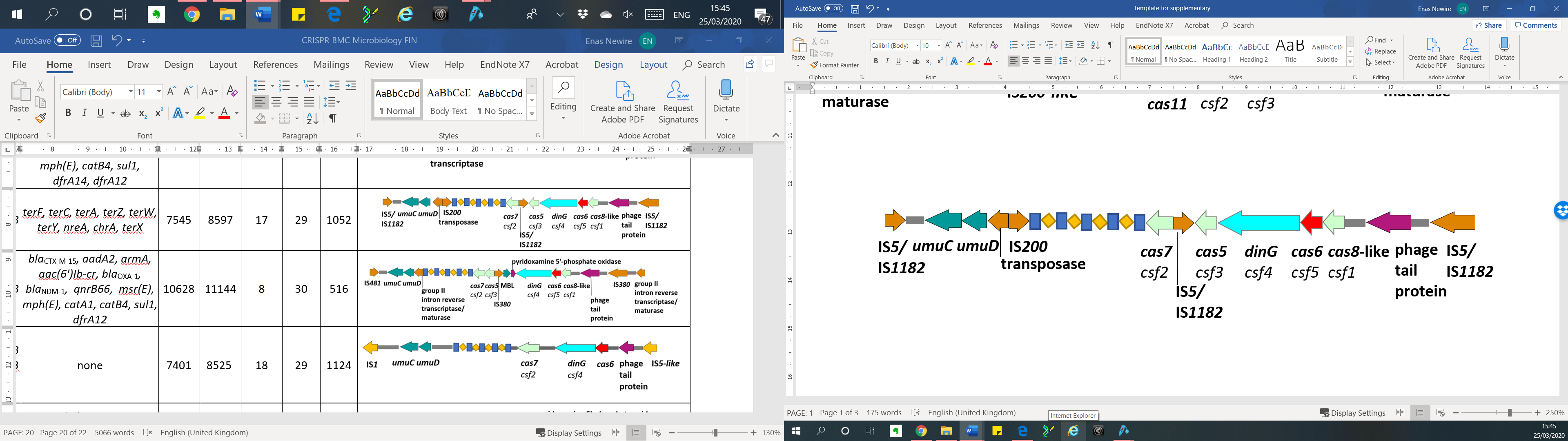 |
| 23 | *K. pneumoniae* strain PMK1 plasmid pPMK1-NDM (CP008933.1) | ST-15 | UK  (2014) | *IncHI1B* | *bla*_CTX-M-15_*, aadA2, armA, aac(6')Ib-cr, bla*_OXA-1_*, bla*_NDM-1_*, qnrB66, msr(E), mph(E), catA1, catB4, sul1, dfrA12* | 10628 | 11144 | 8 | 30 | 516 | 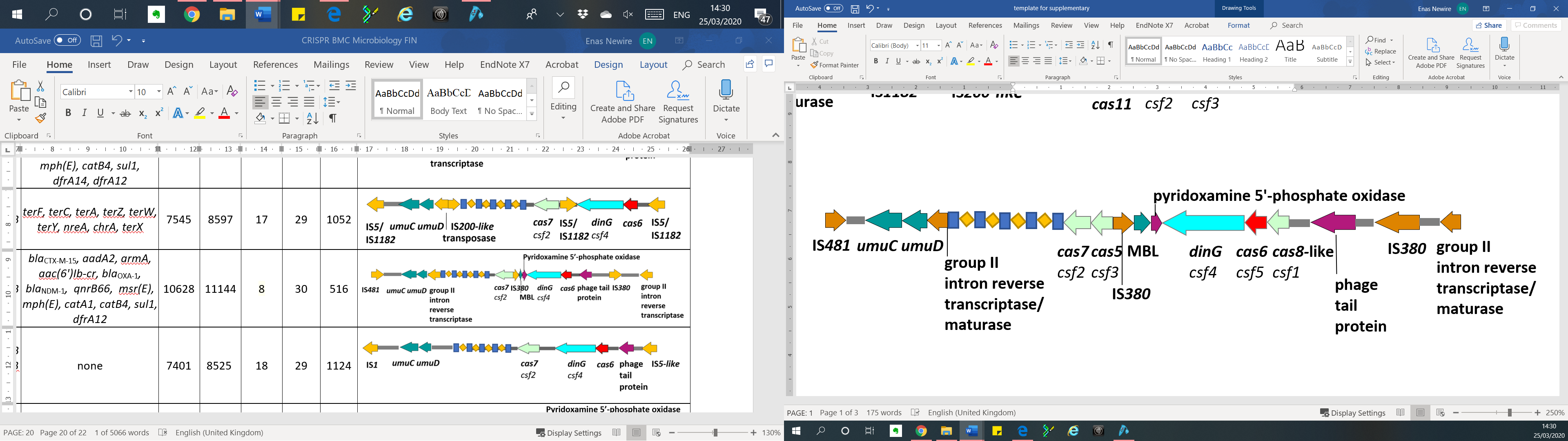 |
| 24 | *K. pneumoniae* strain KPNIH48 plasmid pKPN-edaa (CP026398.1) | ST-252 | USA  (2018) | *IncHI1B/ IncFIB (Mar)* | none | 7401 | 8525 | 18 | 29 | 1124 | 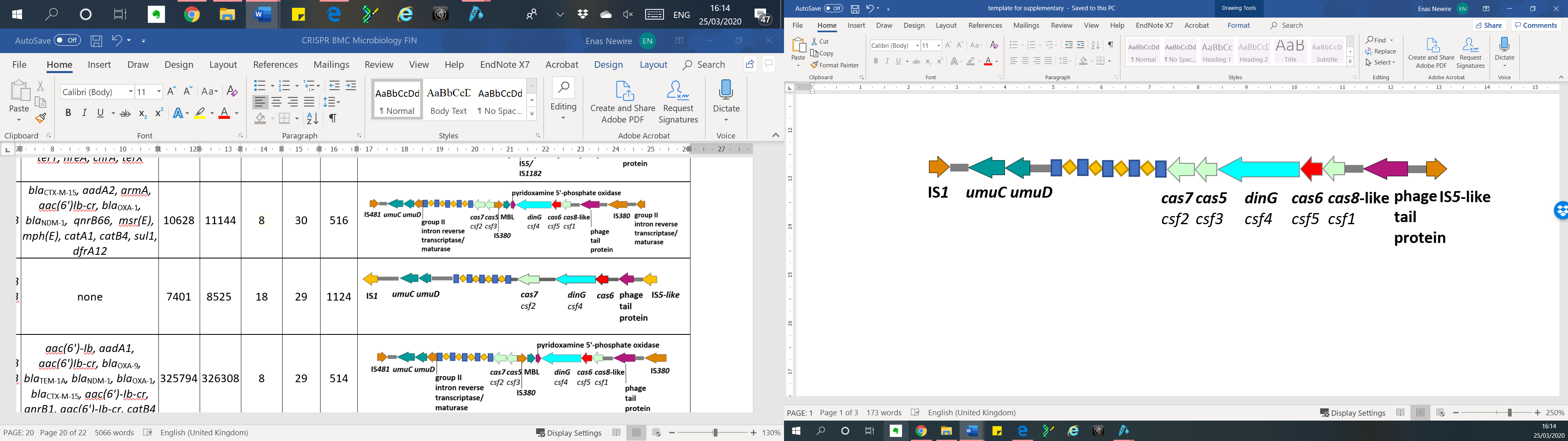 |
| 25 | *K. pneumoniae* strain KPN1481 plasmid pKPN1481-1 (CP020848.1) | ST-906 | USA  (2017) | *IncHI1B/ IncFIB (Mar)* | *aac(6')-Ib, aadA1, aac(6')Ib-cr, bla*_OXA-9_*, bla*_TEM-1A_*, bla*_NDM-1_*, bla*_OXA-1_*, bla*_CTX-M-15_*, aac(6')-Ib-cr, qnrB1, aac(6')-Ib-cr, catB4* | 325794 | 326308 | 8 | 29 | 514 | 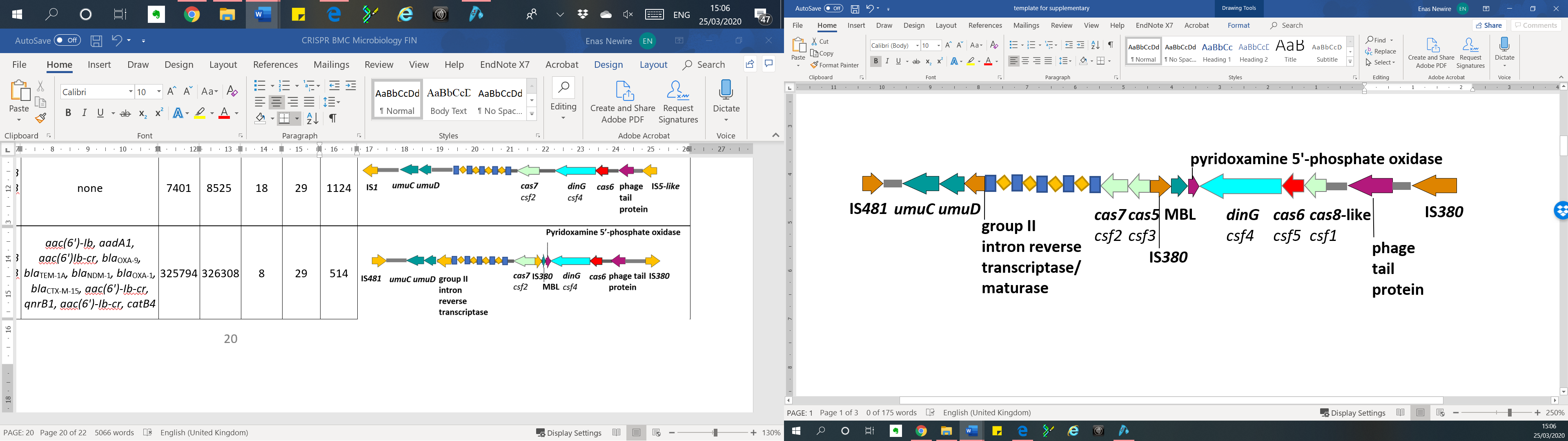 |
| 26 | *K. pneumoniae* strain KSB2_1B plasmid unnamed1 (CP024507.1) | ST-323 | Australia  (2017) | *IncFIB (Mar)* | none | 41987 | 42747 | 12 | 29 | 760 | 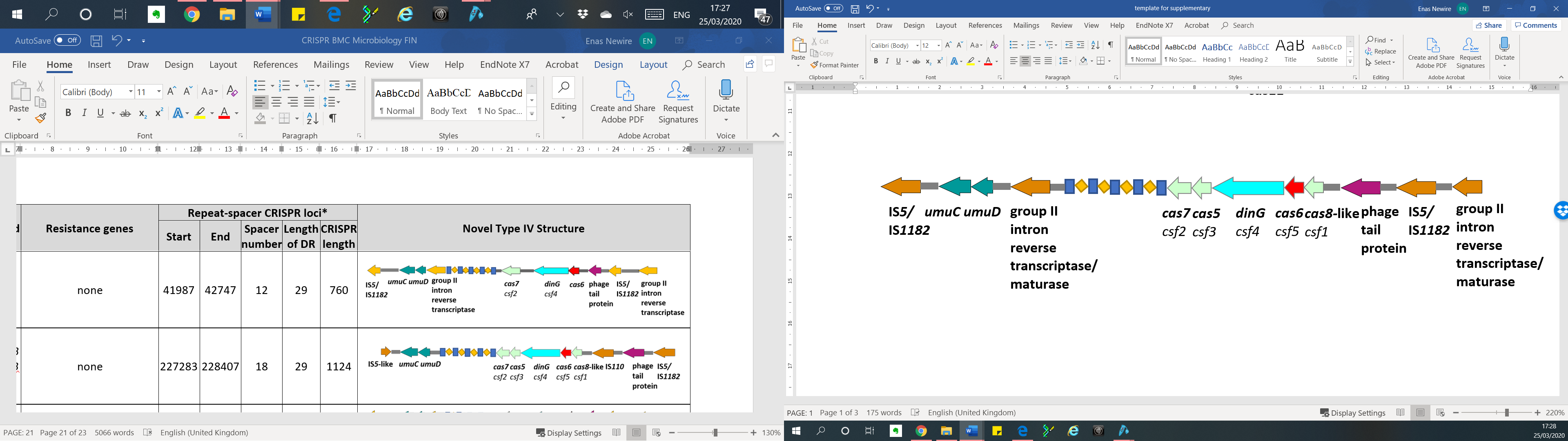 |
| 27 | *K. pneumoniae* strain KPNIH50 plasmid pKPN-bbef (CP026172.1) | ST-252 | USA  (2018) | *IncHI1B/ IncFIB (Mar)* | none | 227283 | 228407 | 18 | 29 | 1124 | 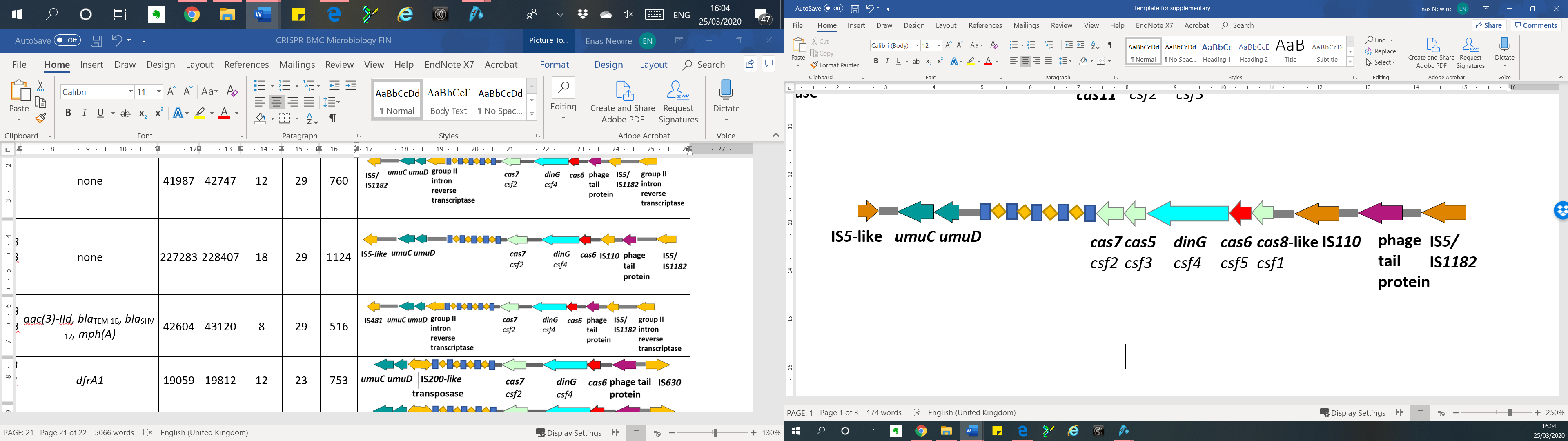 |
| 28 | *K. pneumoniae* strain F44 plasmid p44-1 (CP025462.1) | ST-11 | USA  (2017) | *IncHI1B/ IncFIB (Mar)* | *aac(3)-IId, bla*_TEM-1B_*, bla*_SHV-12_*, mph(A)* | 42604 | 43120 | 8 | 29 | 516 | 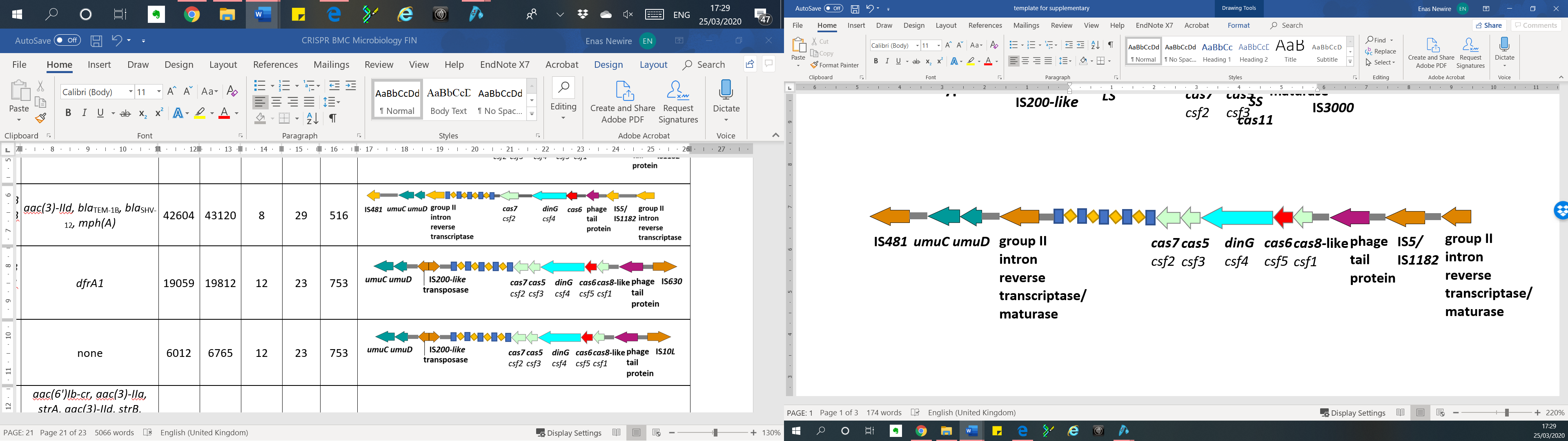 |
| 29 | *K. pneumoniae-53* plasmid1 (SGOL01000000) | ST-502 | Egypt  (2002) | *IncHI1B/ IncFIB (Mar)* | *dfrA1* | 19059 | 19812 | 12 | 23 | 753 | 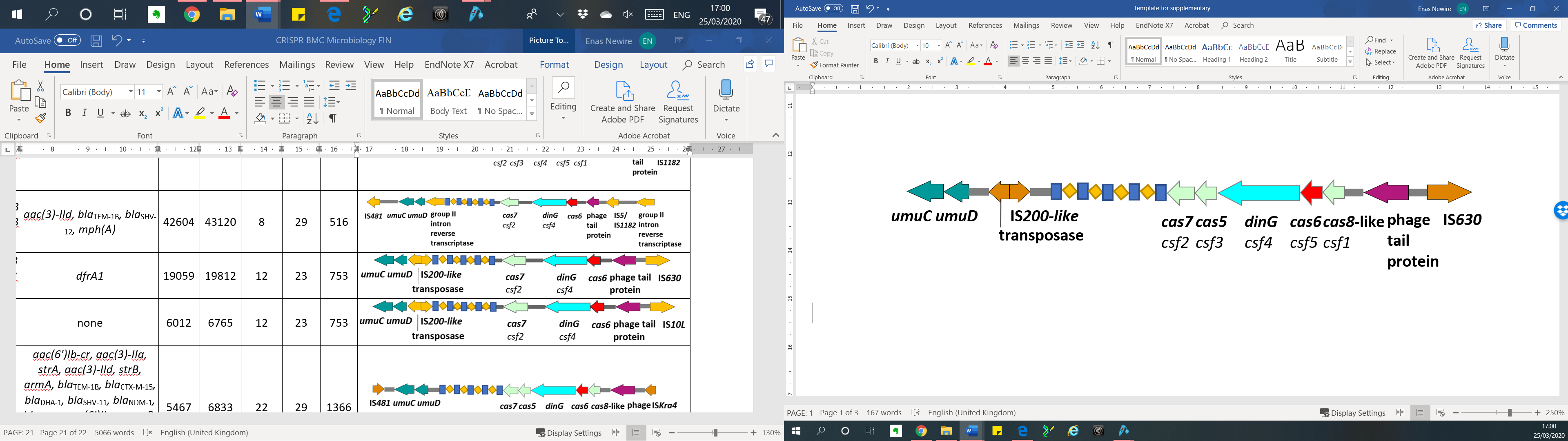 |
| 30 | *K. pneumoniae-65* plasmid 1 (SGOK01000000) | ST-15 | Egypt  (2003) | *IncFIB (Mar)* | none | 6012 | 6765 | 12 | 23 | 753 | 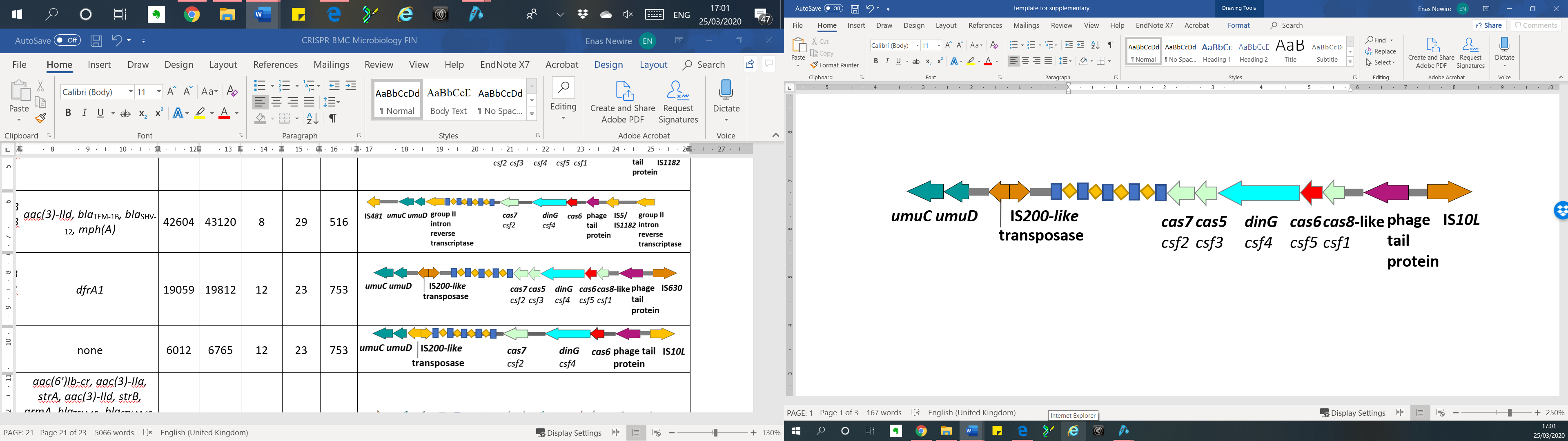 |
| 31 | *K. pneumoniae-CR5*  plasmid 1 (SGOJ01000000) | ST-392 | UK  (2017) | *IncFIB (Mar)* | *aac(6')Ib-cr, aac(3)-IIa, strA, aac(3)-IId, strB, armA, bla*_TEM-1B_*, bla*_CTX-M-15_*, bla*_DHA-1_*, bla*_SHV-11_*, bla*_NDM-1_*, bla*_OXA-1_*, aac(6')Ib-cr, oqxB, oqxA, qnrB66, fosA, msr(E), mph(E), catB4, sul2, sul1, dfrA14* | 5467 | 6833 | 22 | 29 | 1366 | 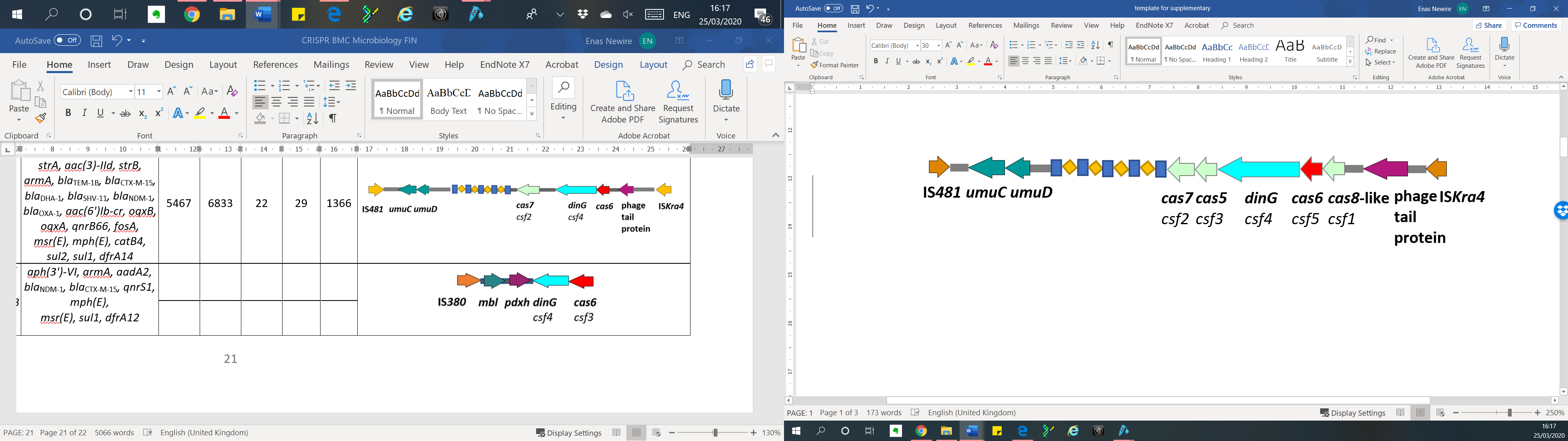 |
| A | *K. pneumoniae* strain K66-45 plasmid pK66-45-1  (CP020902.1) | ST-11 | Norway (2017) | *IncFIB(Mar)/*  *IncHI1B* | *aph(3')-VI, armA, aadA2, bla*_NDM-1_*, bla*_CTX-M-15_*, qnrS1, mph(E),*  *msr(E), sul1, dfrA12* |  |  |  |  |  | 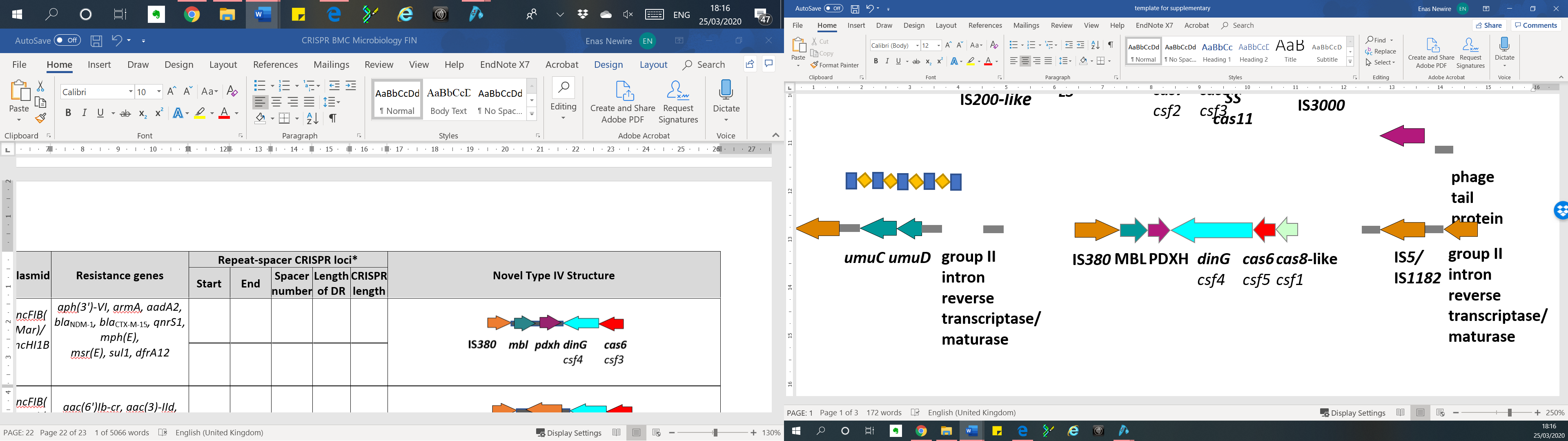 |
| B | *K. pneumoniae* strain AR_0158 plasmid tig00000727  (CP021699.1) | ST-163 | USA (2017) | *IncFIB(Mar)/*  *IncHI1B* | *aac(6')Ib-cr, aac(3)-IId, bla*_OXA-1_*, bla*_SHV-2_*, bla*_NDM-1_*, aac(6')-Ib-cr, qnrB1, catB4, tet(B), dfrA30* |  |  |  |  |  | 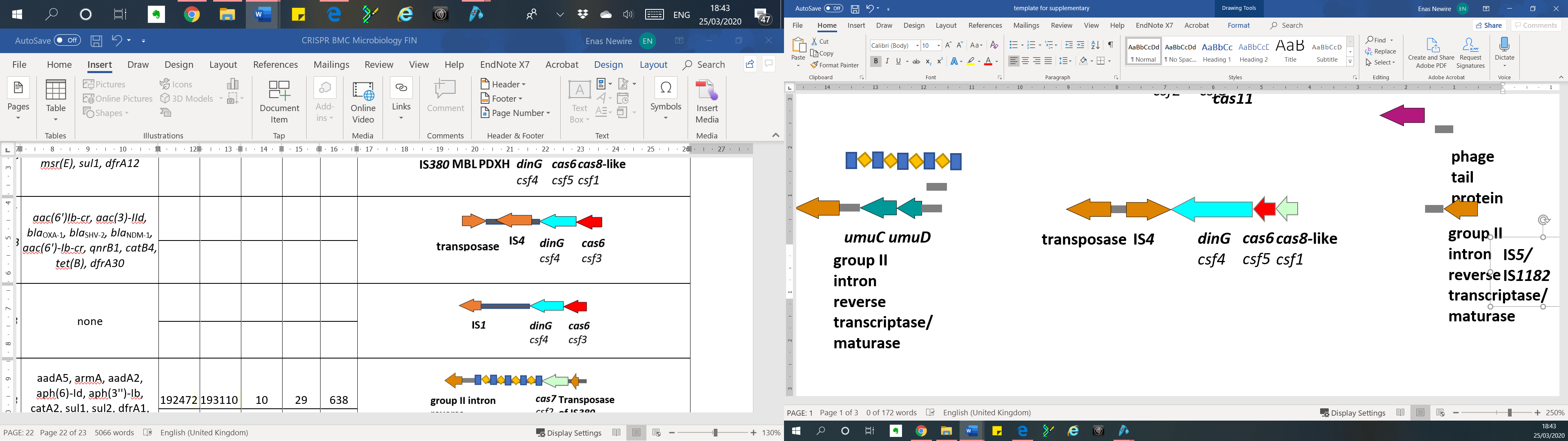 |
| C | *K. pneumoniae* strain LS356 plasmid pKP8-2  (CP025638.1) | ST-485 | China  (2018) | *IncHI1B* | none |  |  |  |  |  | 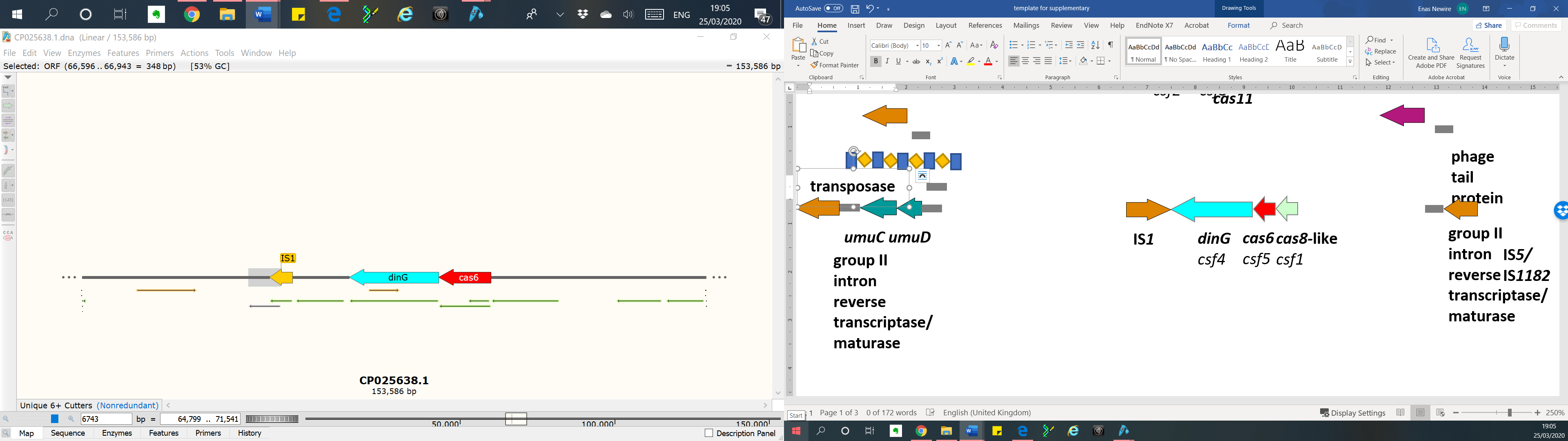 |
| D | *K. oxytoca* strain pKOX3 plasmid p1 ([KY913897.1](https://crispr.i2bc.paris-saclay.fr/cgi-bin/crispr/advRunCRISPRFinder.cgi#KY913897.1_Crispr_1)) |  | China (2017) | *IncHI1B* | aadA5, armA, aadA2, aph(6)-Id, aph(3'')-Ib, catA2, sul1, sul2, dfrA1, dfrA12 | 192472 | 193110 | 10 | 29 | 638 | 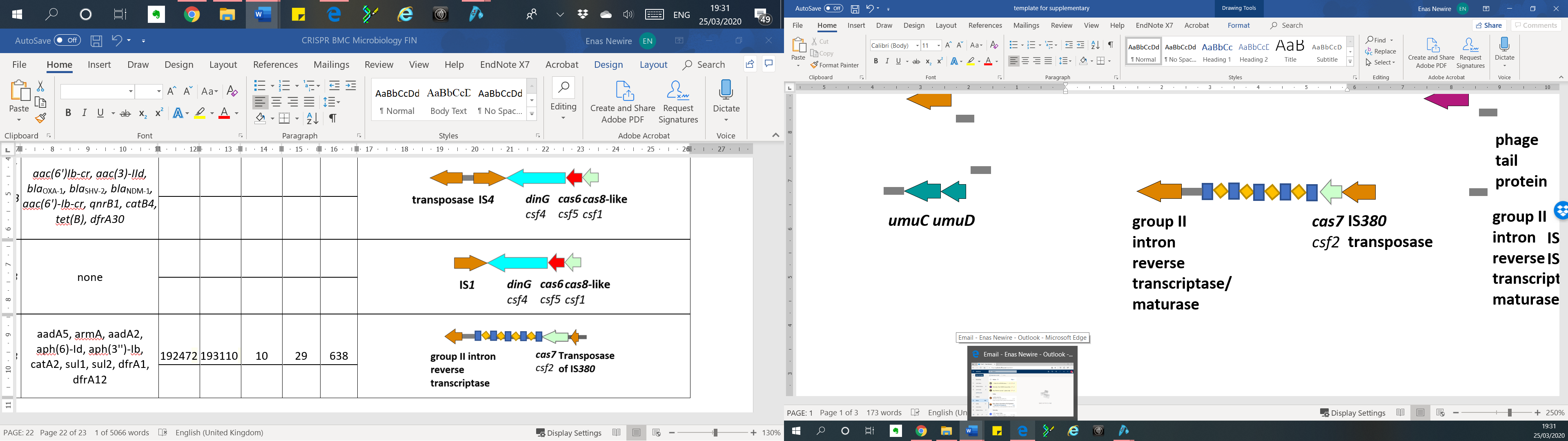 |

† Items 1-31 are the plasmids that carried complete Novel Type IV CRISPR-Cas system. Items A-D represent examples of partial Type IV components that were found carried on the same plasmids.

* All detected repeat-spacer CRISPR were CRISPR1.
